# Hidden genetic diversity in the green alga *Spirogyra* (Zygnematophyceae, Streptophyta)

**DOI:** 10.1186/1471-2148-12-77

**Published:** 2012-06-01

**Authors:** Charlotte Chen, Michael HJ Barfuss, Thomas Pröschold, Michael Schagerl

**Affiliations:** 1 Department of Limnology, University of Vienna, Althanstraße 14, Vienna A-1090, Austria; 2 Department of Systematic and Evolutionary Botany, University of Vienna, Rennweg 14, Vienna, A-1030, Austria; 3 Department Applied Limnology, University of Rostock, Albert-Einstein-Str. 3, Rostock, D-18059, Germany

**Keywords:** Zygnematales, Zygnematophyceae, Non-homoplasious synapomorphy, *Spirogyra*, *Sirogonium*, *Spirotaenia*, SSU rDNA, Diversity

## Abstract

**Background:**

The unbranched filamentous green alga *Spirogyra* (Streptophyta, Zygnemataceae) is easily recognizable based on its vegetative morphology, which shows one to several spiral chloroplasts. This simple structure falsely points to a low genetic diversity: *Spirogyra* is commonly excluded from phylogenetic analyses because the genus is known as a long-branch taxon caused by a high evolutionary rate.

**Results:**

We focused on this genetic diversity and sequenced 130 *Spirogyra* small subunit nuclear ribosomal DNA (SSU rDNA) strands of different origin. The resulting SSU rDNA sequences were used for phylogenetic analyses using complex evolutionary models (posterior probability, maximum likelihood, neighbor joining, and maximum parsimony methods). The sequences were between 1672 and 1779 nucleotides long. Sequence comparisons revealed 53 individual clones, but our results still support monophyly of the genus. Our data set did not contain a single slow-evolving taxon that would have been placed on a shorter branch compared to the remaining sequences. Out of 130 accessions analyzed, 72 showed a secondary loss of the 1506 group I intron, which formed a long-branched group within the genus. The phylogenetic relationship to the genus *Spirotaenia* was not resolved satisfactorily. The genetic distance within the genus *Spirogyra* exceeded the distances measured within any other genus of the remaining Zygnemataceae included in this study.

**Conclusion:**

Overall, we define eight distinct clades of *Spirogyra*, one of them including the genus *Sirogonium*. A large number of non-homoplasious synapomorphies (NHS; 114 NHS in total) was found for *Spirogyra* (41 NHS) and for each clade (totaling 73 NHS). This emphasizes the high genetic diversity of this genus and the distance to the remaining Zygnematophyceae.

## Background

The genus *Spirogyra* is a member of the Zygnemataceae (Zygnematophyceae, Streptophyta). It comprises unbranched, filamentous green algae that are characterized by spirally coiled chloroplasts and sexual reproduction by means of conjugation. *Spirogyra* is commonly found in stagnant or slowly flowing freshwater habitats all over the world [1,2]. It is sometimes referred to as an alga of roadside ditches and is frequently used in introductory biology courses [3] because it often occurs in huge abundances and is easy to address at the generic level. Species definition is mainly based on hypnozygote (also known as zygospores) morphology because the simple morphology in its vegetative state does not permit species recognition. In the latest monograph of *Spirogyra* published by Kadlubowska [4], 386 species are included. They were described using morphological traits, many of them based on a single finding. Ashraf and Godward [5] suggested that the mesospore wall structure analyzed using scanning electron microscopy should be added to the species descriptions because the taxonomy of *Spirogyra* at the light microscopical level remains confusing due to overlapping morphological traits [6]. The morphological species concept, which is also applied in *Spirogyra*, is not proven to represent true biological species, nor does it provide any information on the ecological or genetic diversity in a genus. It also does not elucidate the phylogenetic relationships between taxa [7,8]. Accordingly, the diversity of a genus remains unclear when estimates are based a single species concept. The problems arising for *Spirogyra* from findings without ripe hypnozygotes and the low success rate in inducing conjugation [9-13] call for other ways of addressing the issue of species delimitation and identification.

The Zygnematophyceae (Viridiplantae) represent the most species-rich lineage in the Streptophyta except for the embryophytic land plants [14]. Conjugating green algae including the orders Desmidiales (Desmidiaceae, Peniaceae, Closteriaceae) and Zygnematales (Mesotaeniaceae, Zygnemataceae) form a unique and distinct group. Its taxonomic and phylogenetic separation from other algae is definitive [15-18], but relationships within this group have undergone numerous rearrangements and still remain unclear. The classification schemes within the Zygnematophyceae have been based on morphological traits such as cell size, wall structure, cellular organization or chloroplast structure [1,14,19-21], approaches that have been criticized in the past [22,23]. The Zygnematales are distinguished from the Desmidiales by a smooth cell wall consisting of a single piece, lacking pores and ornamentations [19,21], whereas Desmidiales have cell walls consisting of more than one piece with pores and ornaments [24,25]. Filamentous forms are grouped in the family Zygnemataceae; the unicellular taxa form the family Mesotaeniaceae [4,14,21]. This classification, however, is artificial because polyphyly of both families has been proven by phylogenetic analyses [20,22].

It remains unclear which growth form is primary and which derived [15]. West [26,27] described the ancestral state as filamentous, evolving towards unicellular forms, but Yamagishi [28] stated the opposite. Since the introduction of molecular markers, efforts have been made to solve this question [20,23,29], but the position of the genus *Spirogyra* within the Zygnematophyceae is not fully resolved. The evolutionary rate is one possible reason for this problem: it differs considerably among Zygnemataceaen genera [30]. The uncertain position of *Spirogyra* in phylogenetic analyses is also attributed to the long-branch attraction (LBA) phenomenon [31]. Some genera of the Zygnematophyceae originally defined based on morphology have been revealed as artificial based on molecular markers (e.g., *Staurastrum;*[32]). Furthermore, small subunit nuclear ribosomal DNA (SSU rDNA) phylogenetic studies have often suffered from limited taxon sampling [20]. When genera are represented by just one taxon, authors are unable to address the monophyly of the phylogenetic groups, either at the generic or at higher taxonomic levels. A low number of species within a genus also hinders proving monophyly [33]. In order to assess the monophyly of the genus *Spirogyra* and to investigate whether the low diversity of its vegetative morphology is also reflected in molecular data, we sampled 130 strains of different origin. The position within the Zygnematophyceae and its long-branch position were evaluated by calculating phylogenetic trees with complex evolutionary models. Minimizing the *Spirogyra* LBA problem will also help define phylogenetic relationships among genera. Additionally, we searched for *Spirogyra* taxa with slower evolutionary rates by including isolates from a broad spatial and ecological range and different vegetative morphology. Sampling locations were chosen to cover different types of water bodies in various areas [2]. Morphologically different *Spirogyra* filaments were isolated and cultivated for later use to check if the morphological differences are also reflected in phylogenetic groups. We focused on sampling the morphological diversity of the genus. SSU rRNA was chosen over ribulose-bisphosphate carboxylase large subunit gene (*rbcL*) because other studies already demonstrated the poor resolution of this marker in the Zygnematales and Desmidiales [3,15].

## Results

### Molecular phylogenetic analyses

**Figure 1:**
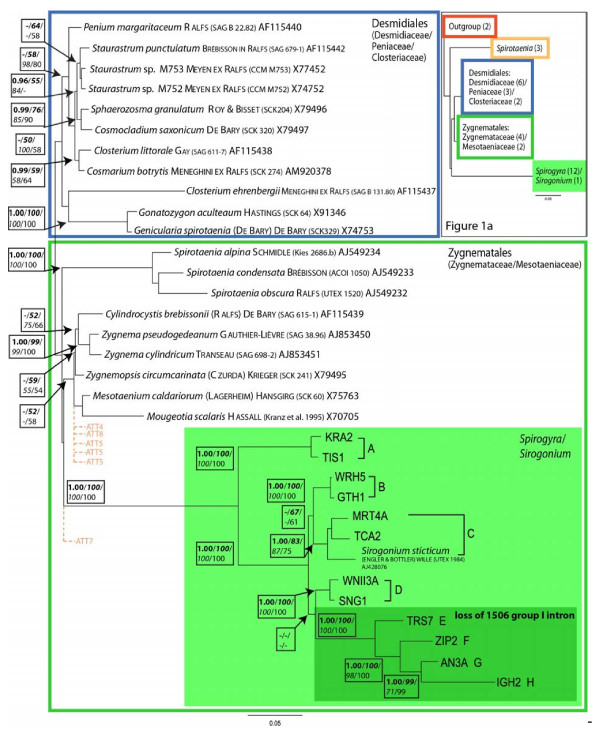
**Combined Zygnematophyceae and *Spirogyra *SSU rRNA phylogeny:** Molecular phylogeny of Desmidiaceae, Peniaceae, Closteriaceae, Mesotaeniaceae and Zygnemataceae based on SSU alignment. The phylogenetic tree was inferred by maximum likelihood analyses of 1720 aligned positions of 33 taxa using PAUP* 4.0b10. TrN+G+I was chosen as best evolutionary model (base frequencies: A 0.25, C 0.23, G 0.27, T 0.25; rate matrix: A-C 1.0000, A-G 1.8721, A-T 1.0000, C-G 1.0000, C-T 4.5252, G-T 1.0000) with the proportion of invariable sites (I= 0.4608) and gamma distribution parameter (G= 0.6376). Posterior Probabilities (>95%; bold; calculated by MrBayes 3.1.2 using the covariation settings (3 million generations, trees from 4100 – 30000)) as well as bootstrap values (>50%) of the maximum likelihood (100 replicates; bold italic), neighbor-joining (1000 replicates; italic), and maximum parsimony (1000 replicates; not bold) are given in the tree (PP/*ML*/*NJ*/MP). No outgroup was used. ATT4, 5, 7 and 8 refer to alternative tree topologies tested with consel – please refer to Table 1 for details. Figure 1a: upper right corner: Combined Zygnematophyceae and *Spirogyra* SSU rRNA phylogeny (same as Figure 1) using 2 taxa as outgroup; numbers in brackets indicate number of taxa included in groups.

**Figure 2:**
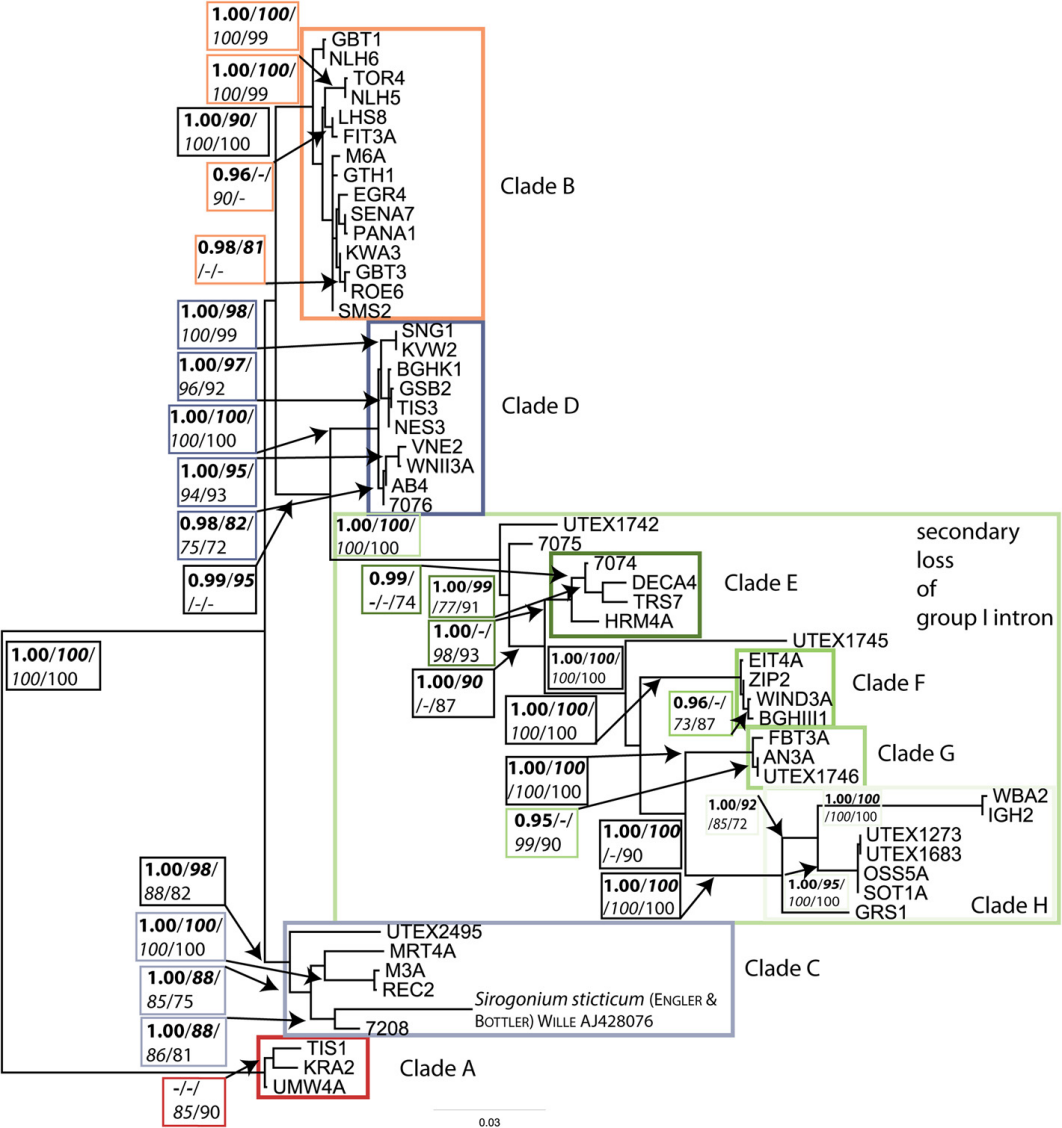
***Spirogyra *SSU rRNA phylogeny:** Unrooted molecular phylogeny of *Spirogyra* based on SSU alignment. Individual clades are highlighted in white boxes; group with secondary loss of group I IC1 intron highlighted by light green-box. The phylogenetic tree was inferred by maximum likelihood analyses of 1645 aligned positions of 55 taxa using PAUP* 4.0b10. GTR+G+I was chosen as best evolutionary model (base frequencies: A 0.23, C 0.24, G 0.29, T 0.24; rate matrix: A-C 1.4341, A-G 2.6641, A-T 1.2357, C-G 1.6993, C-T 5.2526, G-T 1.0000) with the proportion of invariable sites (I= 0.6009) and gamma distribution parameter (G= 0.6856). Posterior Probabilities (>95%; bold; calculated by MrBayes 3.1.2 using the covariation settings (2 million generations, trees from 11070 – 20000)) as well as bootstrap values (>50%) of the maximum likelihood (100 replicates; bold italic), neighbor-joining (1000 replicates; italic), and maximum parsimony (1000 replicates; not bold) are given in the tree (PP/*ML*/*NJ*/MP). No outgroup was used, tree was rooted using clade A.

The 130 *Spirogyra* sequences formed a monophyletic group. This clade, including *Sirogonium*, was subdivided into eight individual sub-clades A to H. Molecular phylogenies were inferred from two data sets, one combining Zygnematophyceae and *Spirogyra* alignment and one alignment comprising only *Spirogyra* and *Sirogonium* sequences (Figures 1, 2). In the phylogeny inferred from the combined Zygnematophyceae alignment (Figure 1), very high bootstrap support was given for branches within the *Spirogyra* clade. Only few branches were without support, one indicating a possible polytomy for clade C and another one indicating the lack of support for phylogenetic resolution between clades B and C, and D to H. Only very closely related taxa received high support by bootstrap values, e.g., the *Zygnema* clade, *Sphaerozosma/Cosmocladium*, *Gonatozygon/Genicularia* and *Closterium/Cosmarium*. The remaining branches lacked support of at least one method. The individual clades of *Spirotaenia* and *Spirogyra* showed very high bootstrap support from all algorithms. The Desmidiales clade was moderately supported (posterior probabilities/maximum likelihood/neighbor joining/maximum parsimony (PP/*ML*/*NJ*/MP): -/*50*/*100*/58); the Zygnematales clade received no support at all. *Sirogonium sticticum* was placed within the *Spirogyra* clade C. None of the algal families analyzed here formed exclusive clades.

When using an outgroup (*Klebsormidium flaccidum* and *Coleochaete scutata*; Figure 1a), the *Spirotaenia* clade was relocated basal to the rest of the Zygnematophyceae; the *Spirogyra*/*Sirogonium* clade formed a sister clade to the remaining Zygnematales and Desmidiales. The overall length of the branches and the classification of taxa to phylogenetic groups did not change. Within the clades, only few rearrangements could be observed in branches that received moderate or poor bootstrap/bayesian support in Figure 1 (data not shown).When testing tree topologies for the combined Zygnematophyceae – *Spirogyra* alignment, the “best tree“ derived from ML analysis (same as phylogeny in Figure 1) was not the overall best tree (Additional file 1: Table S1). The original ML tree (tree 1) and the user defined (UD) tree with *Spirogyra* relocated outside in ancestral position to the clades formed by the Desmidiales and the Zygnematales (tree 2) had the same likelihood and same Bayesian posterior probability values. These two trees were the only ones not rejected by the approximately unbiased test (AU); the tree representing *Spirogyra* as a sister to a Zygnematales clade (including the *Spirotaenia* clade; tree 3) was rejected by all tests except Shimodaira-Hasegawa test (SH) and weighted SH (WSH); *Spirogyra* within the Zygnematales clade (tree 4) was also rejected by all tests except SH. All other trees were significantly worse than the best tree at *p* ≤ 0.05.

In the *Spirogyra* phylogenetic tree (Figure 2), three sequences could not be placed within any clade: UTEX 1742, UTEX 1745 and 7075 share only a small portion of the identification patterns in base composition with adjacent clades. The major clades received very high bootstrap support, except for Clade E that was not supported by ML. The support for branches within clade B was poor due to high sequence similarity. Taxa with a secondary loss of the group I IC1 intron (marked in Figure 1 &2) were clearly separated from the taxa containing the intron (clades A to D with intron; UTEX 1742, 7075, UTEX 1745, clades E to H without intron). The placement of *Sirogonium* in any other clade yielded significantly worse trees in all cases (tested with consel, Additional file 2: Table S2). Also, the relocation of the sequences previously not included in the clades into an adjoining clade was rejected with only one exception: tree 2 (relocation of UTEX 1742 and 7075 into clade E) was not rejected by SH test.

### Sequence similarities in *Spirogyra*

130 *Spirogyra* nuclear encoded SSU rRNA sequences of strains from 79 different sites were sequenced for this study and, in total, 53 different SSU rDNA types (clones) were identified. Thirty sequence types were found once; the remaining 23 were represented by up to 11 accessions. Thirty-eight clones were found only at a single site and 19 more were obtained from up to 6 different sampling sites; 47 sites were represented by just one accession, 32 with up to 10 accessions. Sixty-seven sampling sites exhibited just one clone and 12 had up to 3 different clones.

To describe the genetic variability among the discovered eight lineages (clade A-H; see above), the minimal distance and pair-wise differences were calculated in PAUP. The minimal distance (Table 1, right top) between two clades was 5.09% (clade B and D), the maximum 16.74% (clade A and H), which is 1.57 times higher than the highest value found in the remaining algal groups included in this study. This means that the within-genus difference in *Spirogyra* exceeds the differences among the remaining genera. The highest within-clade distance was observed in clade H (5.17%), followed by clade C (4.31%), whereas the other clades had comparatively low values from 0.25% to 1.23%. Clade A had the lowest distance value to the Zygnemataceae and Mesotaeniaceae used in our analyses (Zygn.: 22.47%; Desm.: 21.44%), while clade H showed the highest distances (Zygn.: 25.63%; Desm.: 25.71%). The pair-wise differences followed the same pattern (Table 1, left bottom) – the biggest difference within *Spirogyra* was recognized between clade A and H (272 nucleotides (nt) difference); the biggest difference exhibited was clade H to the remaining Zygnemataceae (417 nt difference).

**Table 1:** **Table of distances between *Spirogyra *clades; distance measure in the upper right part, pair wise differences in the lower left part**

	**Clade A**	**Clade B**	**Clade C**	**Clade D**	**Clade E**	**Clade F**	**Clade G**	**Clade H**	**Zygn**	**Desm**
**Clade A**	-	11,43	13,46	11,31	12,81	13,79	13,16	16,74	22,47	21,44
**Clade B**	186	-	7,60	5,09	9,21	9,52	9,21	13,51	23,05	22,22
**Clade C**	219	124	-	8,59	11,00	12,04	11,55	15,30	23,23	22,09
**Clade D**	184	83	140	-	8,36	8,91	8,36	13,09	22,76	22,15
**Clade E**	208	150	179	136	-	7,30	6,92	10,78	24,26	23,48
**Clade F**	224	155	196	145	119	-	5,46	9,76	25,31	24,66
**Clade G**	214	150	188	136	113	89	-	8,70	24,86	23,73
**Clade H**	272	220	249	213	176	159	142	-	25,63	25,71
**Zygnematales**	366	375	378	370	395	411	405	417	-	19,49
**Desmidiales**	349	361	359	360	382	400	386	418	319	-
**within clade range**										
**distance**	1,04-1.29	0.06-1.29	0.25-4.56	0.06-1.17	1.17-2.15	0.18-0.31	0.00-0.43	0.06-5.23	1.65-10.63	0.61-10.18
**Pair wise differences**	17-21	1-20	4-61	1-19	19-35	3-5	0-7	0-51	27-173	10-166

### Evolutionary rates in Zygnematophyceae

To test the evolutionary rates among the Zygnematophyceae, the evolutionary models of different data sets were tested by Modeltest. As shown in Table 2, the data sets revealed major differences in base composition. Compared with other Zygnematalean taxa, the G/C content of *Spirogyra* is elevated. Additionally, high variability of sequences is indicated by a lower portion of constant nt and a different pattern in base substitution rates (Table 2). ‘Zygnemataceae + selected *Spirogyra’* and ‘*Spirogyra’* represent the same data sets used for phylogenetic analyses; other data sets were obtained by modifying the previous by exclusion of certain taxa (Table 2). The biggest difference in G/C content (0.0586 units) occurred between the Zygnematophyceae data set excluding *Spirogyra* and the *Spirogyra* data set, pointing out the disparity between the two groups (for respective values see Table 2). Interestingly, the lowest C to T substitution rate was found in the data set used for phylogenetic analyses comprising Zygnematophyceae and selected *Spirogyra* (4.5); the highest value was calculated for the Zygnematophyceae without *Spirogyra* and *Spirotaenia* (7.2). In the *Spirogyra* data set, this value is less elevated (5.3) compared to the other substitution rates. Evolutionary rates were inferred by pair-wise comparison of unambiguously aligned positions of an rRNA SSU alignment of all sequences used in this study in GRate (Table 3 & Additional file 3: Table S3). The comparison was calculated among the genera (Table 3) and among the individual sequences of the alignment (Additional file 3: Table S3). The genus *Spirogyra* showed significant differences to all other Zygnematophyceaen genera (Table 3); the separate clades also revealed highly different values compared to the other Zygnematophyceae (data not shown). For the remaining genera, the picture was less clear – the evolutionary rates of some taxa such as Mesotaeniaceae, Desmidiaceae and Peniaceae did not differ from each other, but did differ from Closteriaceae. Analyses of the individual sequences (Additional file 3: Table S3) revealed that the evolutionary rates of all *Spirogyra* sequences differed significantly from all other Zygnematophyceaen sequences. The one exception was KRA2 from clade A: it differed significantly from all *Spirogyra* sequences, but only from two of the Zygnematophyceaen sequences; all other differences could not be distinguished statistically. Within genera, insignificant values prevailed. The same holds true for *Spirogyra* clades – with one exception: *Spirogyra* clade G showed significant differences among its sequences. Most of the disparities among non-*Spirogyra* sequences were not statistically relevant; differences among the *Spirogyra* sequences of clades B to D were mostly not significant, whereas the differences to the clades E to H (representing accessions not containing the 1506 group I intron) were mostly significant.

**Table 2:** Summary of evolutionary models (chosen by Modeltest) and character states for all individual and combined data sets

	**1.**	**2.**	**3.**	**4.**	**5.**	**6.**
No. Taxa included	33	20	55	13	17	3
Model	TrN+I+G	GTR+I+G	GTR+I+G	GTR+I+G	GTR+I+G	TrN+I+G
-lnL	10368.3486	6458.2769	7311.2354	5725.9985	5393.957	3020.6091
I	0.4608	0.5560	0.6009	0.5228	0.5774	0.8131
G	0.6376	0.7144	0.6856	0.5791	0.6728	-
Base frequencies						
A	0.2545	0.2598	0.2338	0.2436	0.2595	equal rates
C	0.2280	0.2067	0.2439	0.2354	0.2077	
G	0.2665	0.2657	0.2871	0.2808	0.2651	
T	0.2510	0.2678	0.2351	0.2403	0.2677	
G-C	0.4945	0.4724	0.5310	0.5162	0.4728	
Rate matrix						
[A<->C]	1.0000	1.0169	1.4341	1.7371	1.2473	1.0000
[A<->G]	1.8721	1.9209	2.6641	2.7392	1.8624	2.1626
[A<->T]	1.0000	1.2931	1.2357	1.3361	1.5619	1.0000
[C<->G]	1.0000	0.7339	1.6993	1.8149	0.6646	1.0000
[C<->T]	4.5252	5.9738	5.2526	5.9522	7.1560	5.2068
[G<->T]	1.0000	1.0000	1.0000	1.0000	1.0000	1.0000
Character status						
aligned nt	1720	1720	1645	1720	1720	1720
constant nt	1136	1320	1258	1351	1393	1607
MP-informative	479	256	330	269	177	0
MP-uninformative	105	144	57	100	150	113

Data sets used: 1. Zygnematophyceae and 12 *Spirogyra* sequences (same as in combined SSU alignment used in Figure 1). 2. Zygnematophyceae. 3. All *Spirogyra* sequences (same as *Spirogyra* alignment used in Figure 2). 4. 12 *Spirogyra* sequences (same *Spirogyra* sequences as data set 1.). 5. Zygnematophyceae without *Spirotaenia*. 6. *Spirotaenia*.

**Table 3:** **Results of the Relative Rate Test carried out in GRate** [56]**; using only unambiguously aligned positions; not significant: N.S. (*p*** **> 0.05; relative rates not significantly different at 5 % level). Asterisks: *p*** = 0.05 > * > 0.01 > ** > 0.005 > *** (relative rates significantly different)

**taxa**	**Peniaceae**	**Closteriaceae**	**Mesotaeniaceae**	**Zygnemataceae**	**Spirogyra**
Desmidiaceae	***	***	N.S.	N.S.	***
Peniaceae		*	N.S.	*	***
Closteriaceae			*	***	***
Mesotaeniaceae				N.S.	***
Zygnemataceae					***

### Secondary structure and NHS

**Figure 3:**
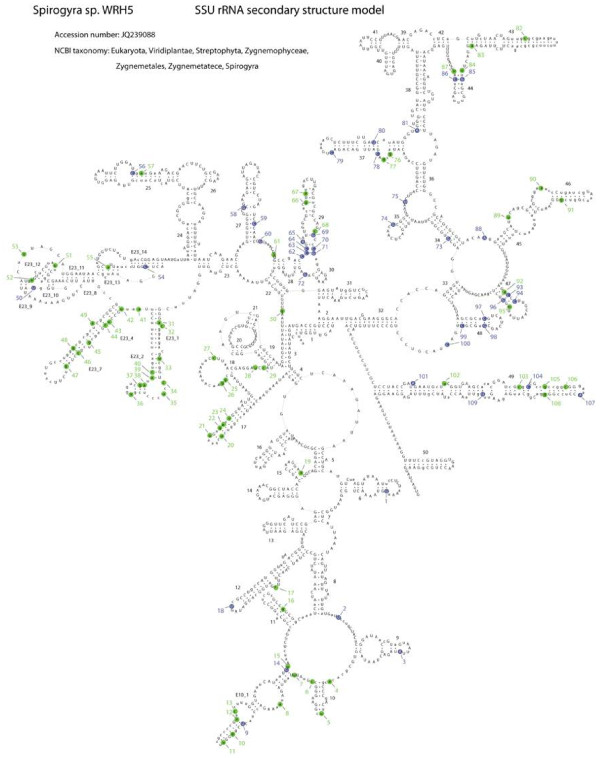
**Putative secondary structure model of the nuclear encoded SSU rRNA of *Spirogyra *sp**.; Strain WRH5 (Acc.No. JQ 239088) from Clade B was chosen as representative sequence; 100% consensus bases for the genus *Spirogyra* are given in upper case letters, variable positions in lower case letters; NHS for *Spirogyra* are marked with blue circles, NHS for one of the clades are marked by green circles, the assigned number indicates the number in the table; see Additional file 4: Table S4 for details.

To discover the variable positions (compensatory base changes (CBCs), hemi-CBCs (HCBCs), and non-homoplasious synapomorphies (NHSs)) in the SSU rDNA of *Spirogyra*, the secondary structures of all strains were compared. No major changes were found in the overall secondary structure of the SSU rDNA (Figure 3). Variable parts (denoted by lower case letters in Figure 3) are predominantly peripheral regions such as E10, E10_1, 17, E23_1, E23_2, E23_4, E23_7, 43, 45, 46, and 49. In an alignment of all Zygnematophyceae and *Spirogyra* sequences used, 114 NHS were identified for the genus *Spirogyra* and/or the individual clades (Additional file 4: Table S4); for the genus *Spirogyra*, 41 NHS were found (blue filled circles in Figure 3). Eight of the NHS were involved in CBCs: the first base pair (bp) in helix 29 (C-G, Nos. 62 and 71), the first C-G pair in helix 44 (Nos. 85 and 86), the fourth bp in helix 47 (C-G, Nos. 93 and 96) and the penultimate bp in helix 48 (C-G, Nos. 97 and 98). NHS for the individual clades were located in the variable parts of the SSU secondary structure, especially in E10_1, E23_1, E23_2, E23_4, E23_7 and 44 (Figure 3, green filled circles). Twenty-nine NHS were found for clade A, 5 for B, 3 for C (including *Sirogonium sticticum*), 8 for D, 3 for E, 9 for F, 4 for G and 12 for H. NHS were also found for groups of clades (data not shown): 23 NHS for clades B to H, 2 NHS for D to H, 16 NHS for the group of taxa with the secondary loss of the group I IC1 intron (clade E to H), 7 for clades F to H and 9 for clades G and H.

## Discussion

We compared 130 SSU rDNA sequences of *Spirogyra* and found a high genetic diversity that was unexpected from the phenotypes. Our phylogenetic analyses revealed that *Spirogyra* splits into eight independent lineages within Zygnematophyceae (clades A-H; Figures 1, 2 and 3). In contrast to low phenotypic and high genetic variability in *Spirogyra*, the genus *Staurastrum* – one the most species-rich genera within the Desmidiaceae comprising around 700 species – showed great variability of morphological characters such as cell shape, size or cell wall ornamentation. In contrast, it yielded no information on phylogenetic relationships or genetic distance, which was interpreted to reflect recent radiation [32]. Nonetheless, even a small number of species and their relatively uniform appearance may not guarantee generic monophyly (e.g., *Spondylosium*, *Desmidium*, *Hyalotheca*, *Netrium*, *Cylindrocystis*, *Mesotaenium*; [33]). Morphology apparently does not reflect genetic diversity in this group. Morphology is even less trustworthy in *Spirogyra*; the genus seems to be very uniform, but the species exhibit a wide overlap of character ranges [2,6,34,35]. Furthermore, changes in ploidy level may occur, also affecting morphology [34,36,37].

The overall phylogenetic relationship of the Zygnematophyceae included in our analysis confirms other studies separating Desmidiaceae, Peniaceae and Closteriaceae from Zygnemataceae and Mesotaeniaceae, although the latter two are not resolved from each other [29,30,38]. Gontcharov et al. [20] already stated that Zygnematales appear to be a polyphyletic assemblage of independent clades. The families Mesotaeniaceae and Zygnemataceae are not monophyletic, proving that the cell wall traits (unornamented, unsegmented) are plesiomorphic [15].

When linking the results of our analysis to rbcL data presented by McCourt [15], the Desmidiales phylogeny is largely congruent, while the Mesotaeniaceae and Zygnemataceae (*sensu* Bold & Wynne [18]) show major differences. In McCourt’s analyses, the branch comprising *Mougeotia/Mesotaenium* and *Cylindrocystis/Zygnemopsis* is placed as an ancestor to other Zygnematophyceae; the branch *Zygnema/Zygogonium* and *Spirogyra*/*Sirogonium*/*Spirotaenia* emerges at a position basal to the Desmidiales. Contrarily, in our phylogenetic tree, the Zygnematales clade is a sister to the Desmidiales clade, although little bootstrap support is given and the genera *Spirogyra*/*Sirogonium* and *Spirotaenia* form individual clades within the Zygnematales. Those clades form distinct branches basal to the remaining Zygnematales branch due to different evolutionary rates of the SSU rDNA.

One reason to choose SSU rDNA over rbcL for phylogenetic analyses is that, as in the rbcL analyses of McCourt [15], phylogenetic relationships among the Desmidiales show rather poor resolution and do not always receive bootstrap support. Furthermore, in the phylogeny in Drummond’s analyses [3], members of the Zygnemataceae exhibit very long branches, whereas members of the genera *Spirogyra* and *Sirogonium* have very short branches indicating only little difference in sequence. This could lead to a misplacement of the genus *Spirogyra*, underestimate diversity in this genus and still yield the LBA problem [31].

The order Zygnematales pools taxa with differing evolutionary rates. On one hand, it is important to include these taxa into phylogenetic analyses to obtain an overview of major relationships. On the other hand, taxa with accelerated evolutionary rates often disturb the analyses. This might be the major reason for low bootstrap support in some of the clades we analyzed. In general, the genetic diversity in the Zygnematales at the generic level has been underestimated in favor of morphological traits that proved to be uninformative at the phylogenetic level [22]. Some genera defined solely by morphological characters are probably artificial and polyphyletic [20]. Thus, the species concept in this group needs urgent revision, and the generic concept requires scrutiny. *Spirogyra* is positioned ancestral to the remaining Zygnematales (except for *Spirotaenia*) in our phylogenetic tree. By testing the UDT against the best tree derived from ML analysis, we conclude that *Spirogyra* has to be placed outside of the Zygnematales clade; this position is definitely not caused by LBA, because no other position in the phylogenetic tree was accepted by UDT testing. Such a position is also supported by analyses of combined Zygnemataceae and *Spirogyra* 1506 group I intron alignments [39].Similar to the rbcL results of McCourt [15], our data do not support the hypothesis of monophyly for groups exhibiting a similar cell shape. In accordance to McCourt’s findings, but in contrast to previous SSU rDNA analyses [29], chloroplast shape seems to be a diagnostic trait: stellate and laminate chloroplast containing taxa form two sister clades [15,40], yet without clear indication of the ancestral chloroplast type [41].

McCourt [15] stated that derived cell and chloroplast forms of placoderm desmids are better photosynthesizers and have achieved greater evolutionary success. This is difficult to reconcile with the ecological success of some of the so-called “primitive forms” such as *Spirogyra*, which is among the most widespread and species-rich conjugating green algae [1]. High evolutionary rates seem to be more common in “primitive forms” such as *Spirogyra*, *Zygnema* and *Mougeotia*, whose evolutionary rates reportedly differ from other Zygnematales [20]. Combined, we conclude that the lack of so-called derived cell and chloroplast forms is compensated for by a high evolutionary rate. This yields a large variety of genotypes and helps cover ecological niches more quickly.

C-U ratios are generally elevated compared to the remaining substitution rates (Table 2). This is because mutations from C to U or U to C in non-coding RNA are not detrimental, as the change in base pairs from G-U or G-C does not affect secondary structure. The biggest difference within the GTR + I + G model occurs between *Spirogyra* and the remaining Zygnematophyceae without *Spirotaenia*: *Spirogyra* shows a 1.9 units lower rate, which partly explains the different mutation rate compared to other algae. Both *Spirogyra* and *Spirotaenia* show a slightly elevated A-G substitution rate (approximately 50% and 16% higher than in the Zygnematophyceae data set, respectively). *Spirogyra* displays not only a higher evolutionary rate, but also a different pattern of base substitution rates compared to the remaining Zygnematales. Evolutionary rates within *Spirogyra* are more diverse than previously expected; significant differences among clades and sequences outside the same clade prevail. Finally, clades B to D, comprising sequences with the 1506 group I intron, form a group with fewer differences compared to clades E to H, comprising taxa without the intron.

The genus *Spirogyra* is clearly monophyletic. No sequences were found that had slower evolutionary rates or that could resolve the long branch reported in previous studies [20]. The individual *Spirogyra* clades found in both of our data sets are essentially the same and well supported by bootstrap and PP values. Moreover, the phylogenetic relationship among those taxa exhibiting the secondary loss of the 1506 group I intron is identical. The *Spirogyra* clade branches are longer than the branches of most genera of Desmidiales. Two long branches within the genus *Spirogyra* are present in both trees; one separates clade A from the rest, and the other separates taxa with the secondary loss of the 1506 group I intron. *Spirogyra* taxa characterized by the absence of the 1506 group I zygnematalean intron form a distinct clade with no exception in both trees. This clearly indicates a single loss event. This explanation is supported by the accelerated evolutionary rate of the exon region of the SSU rDNA [20]. A large number of differentiating NHS exists both within the genus *Spirogyra* (see results) and for each clade. This emphasizes the distinctness and genetic variety within the genus. In contrast, Gontcharov and Melkonian [42] found only very few NHS to circumscribe the different clades in *Cosmarium*.

Earlier hypotheses suggested the unicellular *Spirotaenia* as the ancestor of the filamentous *Spirogyra*[28,40]. Although *Spirotaenia* shares many attributes of the genus *Spirogyra* such as chloroplast shape, absence of the 1506 group I intron [43] and an elevated evolutionary rate, molecular analyses have not proven or clarified its phylogenetic position. Our analyses revealed two well-supported branches at the base of the Zygnematalean clade, but the ancestral form remains unclear. *Spirotaenia*’s unusual position among the Zygnematales was already investigated by Gontcharov and Melkonian [43]. Their results indicate no affiliation of the two genera [43]. They therefore suggested excluding *Spirotaenia* from the Zygnematophyceae sensu stricto. Perhaps the same applies to the genus *Spirogyra,* but this remains to be proven by further genetic analyses. Sequence differences of the unambiguously aligned positions within *Spirogyra* reached 274 nt difference among the strains (a member of clade A and one of clade H), whereas the biggest difference found within the remaining taxa was 247 nt (*Closterium ehrenbergii* and *Spirotaenia obscura*). Also, the average, median and mode of the difference between sequences are higher in *Spirogyra* than in the remaining taxa considered. Within the clade, the range of pair-wise differences within the examined Desmidiales (Desmidiaceae, Peniaceae and Closteriaceae) ranges from 10 to 166 nt: this group includes 3 families and 7 genera. The relative distances within this group do not exceed 10.18. Within the genus *Spirogyra*, 14 individual distances between clades exhibit bigger values (Table 1). The same trend was observed in the remaining Zygnematales (Zygnemataceae and Mesotaeniaceae) – the range of pair-wise differences resulted in 27 to 173 nt, the respective distance values ranged from 1.65 to 10.63. The genetic differences within the genus *Spirogyra*, i.e. among the individual clades, exceed the differences of genera in either of the other groups. This calls for discussion and further investigation on whether *Spirogyra* still should be considered a single genus or rather be subdivided based on the clades we differentiated. This once again underlines the different evolutionary rates and reflects the great genetic variability of the genus *Spirogyra*.

Drummond *et al*. [3] found *Spirogyra* to be monophyletic, but still treated *Sirogonium* as a separate genus based on rbcL data. They were unable to discover morphological characters useful for a generic distinction, simply because the taxa are largely congruent (e.g., number of more or less loosely coiled chloroplasts, reproduction by conjugation and anisogamy of gametangial cells). The diagnostic characters are mainly based on the mode of conjugation: while *Spirogyra* develops conjugation tubes, *Sirogonium* filaments are bent towards each other and form knee-shaped bends, so-called geniculations. Drummond *et al*. [3] also considered the shape and ornamentation of the chloroplast margin as a diagnostic feature, but our observations showed this character to be variable and highly dependent on filament vitality. Other morphological characters such as chloroplast number or cell width are also known to be highly variable and could be explained by polyploidy [1,34-37]. Other authors also found *Spirogyra* and *Sirogonium* forming a single lineage based on single gene analyses (rbcL, SSU rDNA) and combined data sets [15,20,22,43]. Gontcharov stated in 2002 that the genus *Sirogonium* has to be rejected and S. *sticticum* (S. *sticticum* is the type species of the genus) has to be considered as a species within *Spirogyra*[20]. Interestingly, Czurda [10] already suggested including *Sirogonium* into *Spirogyra* as one of four subgenera. We also found *Spirogyra* to be monophyletic and inseparable from *Sirogonium*. Monophyly of the *Spirogyra*/*Sirogonium* clade was determined in all our phylogenetic analyses, placing *Sirogonium* firmly in clade C. All alternative tree topologies relocating *Sirogonium* outside *Spirogyra* were significantly worse than the best tree uniting *Spirogyra* and *Sirogonium*. *Sirogonium* shares NHS signatures with the genus *Spirogyra* and the clade in which it is located.

## Conclusion

*Spirogyra* is monophyletic, incorporating the former genus *Sirogonium*. Genetic diversity and genetic distances within *Spirogyra* exceed the diversity and distances found in other Zygnematophyceaen genera. Our results suggest pursuing the question whether *Spirogyra* should be split into several genera, one of them incorporating *Sirogonium.*

In the surveyed 130 sequences, 53 individually different clones were identified – more than was expected from the simple vegetative morphology. The genus forms eight well-supported clades that differ considerably in NHS pattern – ranging from 3 to 29 NHS for a clade. The genus *Spirogyra* itself exhibits 41 NHS (4 CBCs). Characterizing those clades will require additional studies considering phylogenetic studies on ITS2 secondary structure, hypnozygote morphology, vegetative characteristics and ecology.

## Methods

### Origin of organisms

*Spirogyra* clones used in this study originated from a field survey conducted in 2006 and 2007 [2]. Single filaments were isolated by the author (CC) and incorporated into the Algenkultursammlung Wien (ASW). The non-axenic clones were maintained in 100 ml Erlenmeyer flasks with Desmids medium [44] at 18°C under low light conditions at a 16:8 l:d light cycle (provided by either Philips TLD 36 W/33 or Osram FQ 39 W/840 LUMLUX Cool White). Because only few strains could be identified at species level, cultures were labeled with a code for the corresponding sampling site and date (Additional file 5: Table S5). For our study, we considered 130 *Spirogyra* isolates from different sampling sites and with different vegetative morphologies to cover various ecological niches. Additionally, we included some strains from the UTEX culture collection (UTEX 1746 *Spirogyra pratensis*; UTEX 1273 *S. crassispina*; UTEX 1683 *S. occidentalis*; UTEX1742 *S. juergensis*; UTEX 1745 *S. liana*; UTEX 2495 *S. maxima*).

### DNA extraction

Prior to extraction the cultures were transferred into a defined mineral medium (modified Woods Hole medium; [9]). After 4 to 6 weeks, the algae were harvested with a sterile needle and put into a sterile 2 ml microcentrifuge tube. Samples were frozen at −80°C for at least 4 h and then lyophilized for at least 48 h to improve the DNA yield. Afterwards, the samples were placed in 2 ml Eppendorf tubes containing 5 to 7 glass beads (3 mm diam.) and ground with a homogenizing mill. Total DNA was extracted following a modified CTAB protocol ( [45] modified after [46]).

### DNA amplification and sequencing

Primers used in this study are given in Table 4[47,48]. The PCR reaction mixture was prepared according to the manufacturer’s recommendation. For each PCR reaction, a 10 μl mixture was prepared containing 9 μl ABGene Reddy Mix PCR Master Mix, 0.2 μl for each primer at 20 pM.μ l^-1^, 0.4 μl dimethyl sulfoxide (Sigma) and 0.2 μl DNA template. When the PCR result was unsatisfactory due to low DNA concentration, up to 0.5 μl DNA template was used; when DNA template volume was increased, dimethyl sulfoxide volume was reduced to maintain the total volume of 10 μl, accepting a slight shift in the overall ratio of ingredients. The PCR reaction conditions were an initial hold at 80°C for 5 min followed by 36 cycles starting with a denaturation step at 95°C for 30 s, an annealing step at 55°C for 30 s and an extension step at 72°C for 2 min. A final extension step at 72°C for 8 min and the final hold at 4°C were performed after the 36 cycles were completed. The amplified DNA was cleaned by incubating at 37°C for 45 min, followed by denaturing at 80°C for 15 min together with the enzymes Exonuclease I and Shrimp Alkaline Phosphatase (both from Fermentas) and then subjected to a cycle sequencing reaction. The cycle sequencing reaction conditions were an initial hold at 96°C for 1 min followed by 35 cycles starting with a denaturation step at 96°C for 10 s followed by an annealing step at 50°C for 5 s and an extension step at 60°C for 4 min. The end of the cycles was followed by a final hold at 4°C. Sequencing was performed on a 16-capillary sequencer (Applied Biosystems 3130xl Genetic Analyzer) following the manufacturer’s protocols. The SSU rDNA sequences were used in the phylogenetic analyses; their GenBank accession numbers are given in Additional file 5: Table S5.

**Table 4:** List of primers used in this study

**Primer name**	**Sequence**	**Reference**
EAF3	5'-TCGACAATCTGGTTGATCCTGCCAG-3'	[22]
18sF2	5'-ACCACATCCAAGGAAGGCAGCAG-3'	This study
18sR1	5'-ACGCTATTGGAGCTGGAATTACCGC-3'	This study
18sF3	5'-AGTCCCAACCGTAAACGATGCC-3'	This study
N920R2	5'-CCCTTCCGTCAATTCCTTTAAGTTTC-3'	This study
18sR3	5'-TGTTACGACTTCTCCTTCCTCTAAACG-3'	This study
BR	5'-TTGATCCTTCTGCAGGTTCACCTAC-3'	[23]

### Sequence alignment and phylogenetic analysis

Sequences were aligned manually taking into account the secondary structure of the SSU rDNA [49]. The alignment was refined by comparison of the secondary structure of the sequences. Secondary structure was determined via the Rensselaer bioinformatics web server using mfold [50]. Only unambiguously aligned regions of the sequences were used for analyses; gap-rich regions were excluded. Two different data sets were analyzed: (1) the SSU alignment of 33 Zygnematophyceaen taxa (including 12 *Spirogyra* sequences representing the different clades) and (2) the SSU alignment of 55 *Spirogyra* sequences (clones with identical sequences were represented by only one sequence). The combined Zygnematophyceaen SSU dataset consisted of 1720 unambiguously aligned bases, the *Spirogyra* dataset of 1645 such bases.

The phylogenetic trees presented were inferred by ML settings using PAUP* 4.0b10 [51], and the best models were chosen according to the Akaike Information Criterion by Modeltest 3.7 [52,53]. To test for the best evolutionary model for the analyses, the log-likelihood values of 56 models using Modeltest 3.7 were compared. No outgroup was applied and unrooted phylogenies were used [22]. This was done to avoid LBA *sensu* Philippe [31] caused by unsuitable taxa as outgroups. This approach also follows Gontcharov’s argument that monophyly of the Zygnematophyceae is undoubted but that its position within the Streptophyta is unclear and therefore no suitable outgroup can be chosen. The combined Zygnematophyceaen SSU dataset (1) was also analyzed together with an outgroup (*Klebsormidium flaccidum* and *Coleochaete scutata*) to check for LBA among clades. For the Zygnematophyceaen alignment analyses, the TrN + I + G model was chosen; for the *Spirogyra* alignment the GTR + I + G was chosen by Modeltest. Individual *Spirogyra* clades were labeled with letters A to H in the sequence from basal to derived. The *Spirogyra* alignment was analyzed unrooted to avoid LBA phenomena due to different evolutionary rates [31]. Only individual sequences were used for analyses to reduce computational effort. Bayesian inference (PP) was calculated using MrBayes 3.1.2. [54,55] using 3 million generations, sampling every 100 generations and MCMC chains = 4. All trees below the burnin value of 0.01 were discarded as burnin, the consensus tree was calculated using PAUP*. The robustness of the trees was assessed by bootstrap support values. These were calculated using the corresponding evolutionary model chosen by Modeltest by ML (100 replicates), distance NJ (1000 replicates), and MP (1000 replicates) methods using the accordant settings/evolutionary model for each dataset. Insignificant values were not included in figures (PP < 0.95, ML, NJ, MP < 50%). Details of the corresponding evolutionary models and Bayesian analyses are given in the legend of the accordant figures and in Table 2.

Additionally, a distance matrix was calculated using PAUP* to evaluate the genetic distances among the *Spirogyra* clades (Table 1). The value was obtained by dividing the number of differing bases by the number of total (=aligned) bases. Values were transformed to % values, so numbers near 0 indicate a high identity or short distance; values approximating 100 indicate low similarity and large distance. Relative rate tests were carried out among all genera and for all accessions individually used for phylogeny in GRate 0.4 ( http://bioinfweb.info/Software/GRate; [56]).

### Tree topology tests

UD trees were generated manually based on the “best tree” (derived from ML analysis; same topology as ML phylogeny used for Figure 1) using TreeView 1.6.6 [57]. To compare the UD-trees with the “best tree”, the alignment was loaded into PAUP and site-wise log-likelihood values for each tree were calculated. The result was used as input for the program CONSEL v0.1 k [58], calculating probability values according to KH [59], SH ( [60], both weighted = w and unweighted), and AU using the multiscale bootstrap technique [61] (Additional file 1: Table S1 and Additional file 2: S2).

### Apomorphy analysis

The secondary structure of the SSU rDNA (Figure 3) was modeled after Wuyts [49], following the same numbering pattern. To find all NHS, the method described by Marin et al. [62] was applied. To identify genetic characteristics for the different groups (synapomorphic signatures; [63]), the secondary structure in an alignment of Zygnematophyceae and all sequenced *Spirogyra* clones was compared, and NHS and CBCs were determined according to Marin et al. [62]. The analysis was performed with two aims: (1) to find NHS for the genus *Spirogyra* and (2) to identify NHS for each individual clade within the genus *Spirogyra*. For both, the two NHS criteria were applied: (1) absence of convergent evolution outside the clade and (2) strict conservation within the clade (Additional file 4: Table S4).

## Abbreviations

ATT: alternative tree topology tested; AU: approximately unbiased test; Bp: base pair; (H-) CBC (Hemi-): Compensatory Base Change; KH: Kishino-Hasegawa test; LBA: long branch attraction; ML: maximum likelihood; MP: maximum parsimony; NHS: Non-Homoplasious Synapomorphy; NJ: neighbor joining; nt: nucleotide; PP: posterior probability; RbcL: ribulose-bisphosphate carboxylase large subunit gene; rDNA: nuclear ribosomal DNA; SH: Shimodaira-Hasegawa test; SSU: small subunit; UD: tree user defined tree; WSH: weighted SH.

## Authors’ contributions

CC co-designed the study, collected the samples, generated the sequence data, did the analyses and prepared the manuscript. MHJB helped with the lab work, obtaining and analyzing the data. TP provided conceptual guidance and supported the data analysis. MS designed the study and provided conceptual support. All authors contributed to the preparation of the manuscript, and read and approved the final version.

## Supplementary Material

Additional file 1 **Table S1.** Comparison of the maximum likelihood tree (Zygnematophyceaen alignment) with user defined trees by AU (P-value of the approximately unbiased test calculated from multiscale bootstrap), PP, KH, SH and weighted SH. Trees significantly worse than the best trees at p≤ 0.05 are indicated by grey highlighting. Click here for file

Additional file 2 **Table S2.** Comparison of the maximum likelihood tree (*Spirogyra* alignment) with user defined trees by AU, KH, SH and weighted SH. Trees significantly worse than the best trees at p≤ 0.05 are indicated by grey highlighting. Click here for file

Additional file 3 **Table S3.** Results of the Relative Rate Test carried out in GRate [56]; using only unambiguously aligned positions of all sequences used in this study; not significant: N.S. (*p* > 0.05; relative rates not significantly different at 5% level). Asterisks: *p* = 0.05 > * > 0.01 > ** > 0.005 > *** (relative rates significantly different). Click here for file

Additional file 4 **Table S4.** List of NHS found for the genus *Spirogyra* and clades. Click here for file

Additional file 5 **Table S5.** Origin of *Spirogyra* isolates. Click here for file
