# Supplementary material for: Hidden genetic diversity in the green alga Spirogyra (Zygnematophyceae, Streptophyta)
Source: BMC Evol Biol. 2012 Jun 1;12:77. doi: 10.1186/1471-2148-12-77 (PMC3527229; doi:10.1186/1471-2148-12-77)
Supplement: Additional file 1 — Table S1. Comparison of the maximum likelihood tree (Zygnematophyceaen alignment) with user defined trees by AU (P-value of the approximately unbiased test calculated from multiscale bootstrap), PP, KH, SH and weighted SH. Trees significantly worse than the best trees at p≤ 0.05 are indicated by grey highlighting. [file 1471-2148-12-77-S1.doc]

Table 5: List of NHS found for the genus *Spirogyra* and clades

| **Number** |  |  |  |  |  |  |  |  |  |  |  |  |  |  |  |  |  |  |  |  |  |  |  |  |  |  |  |  |  |  |  |
| --- | --- | --- | --- | --- | --- | --- | --- | --- | --- | --- | --- | --- | --- | --- | --- | --- | --- | --- | --- | --- | --- | --- | --- | --- | --- | --- | --- | --- | --- | --- | --- |
| *Spirogyra* | 1 | 2 | 3 |  |  |  |  |  | 9 |  |  |  |  | 14 |  |  |  | 18 |  |  |  |  |  |  |  |  |  |  |  |  |  |
| Clade A |  |  |  |  |  |  | 7 | 8 |  |  |  | 12 |  |  |  | 16 |  |  | 19 |  |  |  |  |  |  |  | 27 |  | 29 | 30 | 31 |
| Clade B |  |  |  |  |  |  |  |  |  |  |  |  |  |  |  |  |  |  |  |  |  |  |  |  |  |  |  |  |  |  |  |
| Clade C |  |  |  |  |  |  |  |  |  |  |  |  |  |  |  |  |  |  |  |  |  |  |  |  |  |  |  |  |  |  |  |
| Clade D |  |  |  |  |  |  |  |  |  |  |  |  | 13 |  |  |  | 17 |  |  | 20 |  |  |  |  |  | 26 |  | 28 |  |  |  |
| Clade E |  |  |  |  |  |  |  |  |  |  |  |  |  |  |  |  |  |  |  |  |  |  |  | 24 |  |  |  |  |  |  |  |
| Clade F |  |  |  |  |  | 6 |  |  |  | 10 |  |  |  |  |  |  |  |  |  |  | 21 | 22 | 23 |  |  |  |  |  |  |  |  |
| Clade G |  |  |  |  |  |  |  |  |  |  | 11 |  |  |  |  |  |  |  |  |  |  |  |  |  |  |  |  |  |  |  |  |
| Clade H |  |  |  | 4 | 5 |  |  |  |  |  |  |  |  |  | 15 |  |  |  |  |  | 21 |  |  |  | 25 |  |  |  |  |  |  |
| **Position in** | 0 | 0 | 0 | 0 | 0 | 0 | 0 | 0 | 0 | 0 | 0 | 0 | 0 | 0 | 0 | 0 | 0 | 0 | 0 | 0 | 0 | 0 | 0 | 0 | 0 | 0 | 0 | 0 | 0 | 0 | 0 |
| **Alignment** | 0 | 1 | 1 | 1 | 2 | 2 | 2 | 2 | 2 | 2 | 2 | 2 | 2 | 2 | 2 | 3 | 3 | 3 | 4 | 5 | 5 | 5 | 5 | 5 | 5 | 5 | 5 | 5 | 5 | 6 | 6 |
|  | 9 | 4 | 7 | 9 | 0 | 1 | 1 | 2 | 3 | 4 | 4 | 5 | 6 | 8 | 8 | 1 | 5 | 6 | 5 | 2 | 2 | 3 | 3 | 3 | 5 | 5 | 6 | 7 | 7 | 4 | 7 |
|  | 1 | 0 | 0 | 1 | 2 | 4 | 9 | 8 | 9 | 2 | 5 | 9 | 3 | 2 | 3 | 4 | 1 | 5 | 2 | 1 | 7 | 0 | 1 | 3 | 0 | 1 | 3 | 5 | 7 | 0 | 7 |
| **Helix number** | 6 | J8-9 | L9 | J9-10 | L5 | J5-E10_1 | J5-E10_1 | BE10_1 | E10_1 | E10_1 | E10_1 | E10_1 | BE10_1 | E10_1 | JE10_1-11 | 11 | B12 | L12 | B19 | B17 | L17 | 17 | 17 | 17 | L18 | L18 | L18 | J18-19 | J18-19 | J3-22 | E23_1 |
| without : helix, J: link, L: endloop, B: buldge | | | | | |  |  |  |  |  |  |  |  |  |  |  |  |  |  |  |  |  |  |  |  |  |  |  |  |  |  |
| D13324 | U | A | C | A | C | C | U | A | C | G | U | A | ~ | A | A | G | C | A | A | U | A | U | C | U | A | C | C | U | A | G | U |
| X74000 | U | A | C | A | C | C | U | A | C | G | U | A | ~ | A | A | G | C | A | A | U | A | U | C | U | A | C | C | U | A | G | U |
| AF115438 | U | A | C | A | C | U | U | A | C | A | U | G | U | A | A | G | C | A | A | U | G | U | C | U | A | C | C | U | C | G | U |
| AF115437 | C | G | C | U | C | U | U | C | G | G | C | G | U | A | A | G | C | A | A | U | G | U | C | U | C | G | C | U | A | G | G |
| AF115442 | U | A | C | A | C | C | U | A | C | A | U | G | U | A | A | G | C | A | A | C | G | U | U | U | A | C | C | U | C | G | U |
| X91346 | U | A | C | A | U | U | U | A | C | G | U | G | U | A | A | G | C | A | A | U | G | U | C | C | A | C | C | U | C | G | U |
| AF115440 | U | A | C | A | U | C | U | A | C | A | U | G | U | A | A | G | C | A | A | U | G | U | C | U | A | C | C | U | C | G | U |
| X79496 | U | A | C | A | C | C | U | A | C | A | U | G | U | A | A | G | C | A | A | U | G | U | C | U | A | C | C | U | C | G | U |
| X77452 | U | A | C | A | C | U | U | A | C | A | U | G | U | A | A | G | C | A | A | U | G | U | U | U | A | C | C | U | C | G | U |
| X74752 | U | A | C | A | C | C | U | A | C | A | U | G | U | A | A | G | C | A | A | U | G | U | U | U | A | C | C | U | C | G | U |
| AF115439 | U | A | C | A | C | C | U | A | G | G | U | G | U | A | A | G | C | A | A | U | G | U | C | A | A | C | C | C | A | G | U |
| AM920378 | U | A | C | A | C | C | U | A | C | G | U | G | U | A | A | G | C | A | A | U | G | U | C | U | A | C | C | U | C | G | U |
| X75763 | U | A | C | A | U | C | U | A | G | G | U | G | U | A | A | G | ~ | A | A | U | G | U | C | U | A | C | C | C | C | G | C |
| X74753 | U | A | C | A | U | U | U | A | C | G | U | G | U | A | A | G | C | A | A | U | G | U | C | C | A | C | C | U | C | G | U |
| X79495 | U | A | C | A | U | C | U | A | G | G | U | G | U | A | A | G | C | A | A | U | G | U | C | U | A | C | C | C | C | G | U |
| X79497 | U | A | C | A | C | C | U | A | C | A | U | G | U | A | A | G | C | A | A | U | G | U | C | U | A | C | C | U | C | G | U |
| AJ853450 | U | A | C | A | U | C | U | A | G | G | U | G | U | A | A | G | C | A | A | U | G | U | C | ~ | A | C | C | C | C | G | U |
| AJ853451 | U | A | C | A | C | C | U | A | G | G | U | G | U | A | A | G | C | A | A | U | G | U | C | U | A | C | C | C | C | G | U |
| AJ549234 | N | A | C | A | U | U | U | A | G | G | C | G | C | A | A | G | C | A | A | U | G | U | C | U | A | C | C | U | C | G | U |
| AJ549233 | U | A | C | A | U | U | U | A | G | G | U | G | C | A | A | G | C | A | A | U | G | U | C | A | A | C | C | U | C | G | U |
| AJ549232 | U | A | C | A | C | U | U | A | G | G | U | G | U | A | A | G | C | A | A | U | G | U | C | U | A | C | C | ~ | A | G | U |
| X70705 | U | A | C | U | A | U | U | A | G | G | U | G | U | A | A | G | ~ | A | A | U | G | U | C | U | A | C | C | U | C | G | C |
| KRA2_A | G | U | A | A | A | U | G | U | U | G | U | U | U | G | ~ | U | C | U | U | U | G | A | U | U | G | G | U | U | U | A | G |
| TIS1_A | G | U | A | A | A | U | G | U | U | G | U | U | U | G | ~ | U | C | U | U | U | G | A | U | U | G | G | U | U | U | A | G |
| WRH5_B | G | U | A | A | C | C | U | A | U | G | U | G | C | G | U | G | C | U | A | U | G | A | C | U | G | G | C | U | C | G | A |
| GTH1_B | G | U | A | A | C | C | U | A | U | G | U | G | C | G | U | G | C | U | A | U | G | A | C | U | G | G | C | U | C | G | A |
| MRT4A_C | G | U | A | A | C | C | U | A | U | G | U | G | C | G | U | G | C | U | A | U | G | A | C | U | G | G | C | U | C | G | A |
| TCA2_C | G | U | A | A | C | C | U | A | U | G | U | A | C | G | U | G | C | U | A | U | G | A | C | U | G | G | C | U | C | G | A |
| AJ428076 | G | U | A | A | C | U | U | A | U | G | U | A | U | G | U | G | C | U | A | U | G | A | C | U | G | G | C | U | C | G | A |
| WNII3A_D | G | U | A | A | C | C | U | A | U | G | C | G | A | G | U | G | U | U | A | A | G | A | C | U | G | U | C | A | C | G | A |
| SNG1_D | G | U | A | A | C | C | U | A | U | G | U | G | A | G | U | G | U | U | A | A | G | A | U | U | G | U | C | A | C | G | A |
| TRS7_E | G | U | A | C | C | C | U | A | U | G | C | A | C | G | U | G | C | U | A | U | G | A | C | G | G | G | C | U | C | G | A |
| ZIP2_F | G | U | A | C | C | A | U | A | U | C | ~ | A | C | G | U | G | C | U | A | U | U | G | A | U | G | G | C | U | C | G | A |
| AN3A_G | G | U | A | C | C | C | U | C | U | G | G | A | C | G | U | G | C | U | A | U | A | C | G | U | G | G | C | U | C | G | C |
| IGH2_H | G | U | A | G | G | U | U | C | U | G | U | A | C | G | C | G | C | U | A | U | C | C | G | U | U | G | C | U | C | G | A |

Table 5: List of NHS found for the genus *Spirogyra* and clades (continued)

| **Number** |  |  |  |  |  |  |  |  |  |  |  |  |  |  |  |  |  |  |  |  |  |  |  |  |  |
| --- | --- | --- | --- | --- | --- | --- | --- | --- | --- | --- | --- | --- | --- | --- | --- | --- | --- | --- | --- | --- | --- | --- | --- | --- | --- |
| ***Spirogyra*** |  |  |  |  |  |  |  |  |  |  |  |  |  |  |  |  |  |  | 50 |  |  |  | 54 |  | 56 |
| Clade A | 32 |  |  |  |  |  | 38 | 39 | 40 | 41 |  |  | 44 | 45 |  |  |  | 49 |  | 51 | 52 | 53 |  |  |  |
| Clade B |  |  |  |  |  |  |  |  |  |  |  |  |  |  |  |  |  |  |  |  |  |  |  | 55 |  |
| Clade C |  |  |  |  |  |  |  |  |  |  |  |  | 44 |  |  |  |  |  |  |  |  |  |  |  |  |
| Clade D |  |  |  |  |  |  |  |  |  |  |  | 43 |  |  |  |  |  |  |  |  |  |  |  |  |  |
| Clade E |  |  |  |  | 36 |  |  | 39 |  |  |  |  |  |  |  |  |  |  |  |  |  |  |  |  |  |
| Clade F |  |  |  |  |  |  |  |  | 40 |  |  |  |  |  |  | 47 |  |  |  |  |  |  |  |  |  |
| Clade G |  |  | 34 | 35 |  |  |  |  |  |  |  |  |  |  |  |  |  |  |  |  |  |  |  |  |  |
| Clade H |  | 33 |  |  |  | 37 |  |  |  | 41 | 42 |  |  |  | 46 |  | 48 |  |  |  |  |  |  |  |  |
| **Position in** | 0 | 0 | 0 | 0 | 0 | 0 | 0 | 0 | 0 | 0 | 0 | 0 | 0 | 0 | 0 | 0 | 0 | 0 | 0 | 0 | 0 | 0 | 0 | 0 | 0 |
| **Alignment** | 6 | 6 | 6 | 6 | 7 | 7 | 7 | 7 | 7 | 7 | 7 | 7 | 7 | 7 | 7 | 7 | 7 | 7 | 8 | 8 | 8 | 8 | 8 | 8 | 9 |
|  | 7 | 8 | 9 | 9 | 0 | 0 | 0 | 0 | 1 | 4 | 4 | 5 | 5 | 5 | 6 | 6 | 8 | 8 | 1 | 3 | 4 | 5 | 7 | 9 | 7 |
|  | 8 | 6 | 1 | 3 | 1 | 6 | 7 | 9 | 0 | 3 | 6 | 0 | 1 | 6 | 1 | 5 | 0 | 7 | 5 | 3 | 5 | 1 | 8 | 2 | 9 |
| **Helix number** | BE23_1 | E23_2 | BE23_2 | BE23_2 | LE23_2 | E23_2 | E23_2 | E23_2 | E23_2 | JE23_1-E23_4 | E23_4 | BE23_4 | E23_4 | E23_4 | E23_4 | E23_4 | E23_4 | BE23_4 | E23_10 | LE23_11 | JE23_12-E23_9 | JE23_12-E23_9 | E23_14 | E23_13 | 25 |
| without : helix, J: link, L: endloop, B: buldge | | | | |  |  |  |  |  |  |  |  |  |  |  |  |  |  |  |  |  |  |  |  |  |
| D13324 | G | U | C | G | C | U | G | A | C | U | U | G | G | G | C | G | G | A | G | U | A | U | U | U | U |
| X74000 | G | U | C | G | C | U | G | A | C | U | U | G | G | G | C | G | G | A | G | U | A | U | U | U | U |
| AF115438 | U | U | C | G | ~ | G | A | A | C | U | U | G | G | G | C | A | G | A | G | U | A | U | U | G | U |
| AF115437 | U | U | C | G | C | G | A | A | C | U | U | G | G | G | C | G | G | G | G | ~ | A | U | U | G | U |
| AF115442 | U | U | C | G | C | G | A | A | C | U | U | G | G | G | U | G | G | A | G | U | A | U | U | G | U |
| X91346 | U | U | C | G | U | G | A | A | C | U | U | G | G | A | U | A | A | A | G | U | A | U | U | G | U |
| AF115440 | U | U | C | G | ~ | G | A | A | C | U | U | G | G | G | U | G | G | A | G | U | A | U | U | G | U |
| X79496 | U | U | C | G | C | G | A | A | C | U | U | G | G | G | C | G | G | A | G | U | A | U | U | G | U |
| X77452 | U | U | C | G | C | G | A | A | C | U | U | G | G | G | U | A | A | A | G | U | A | U | U | G | U |
| X74752 | U | U | C | G | C | G | A | A | C | U | U | G | G | G | C | G | G | A | G | U | A | U | U | G | U |
| AF115439 | U | U | C | G | U | G | A | A | C | U | U | G | G | G | U | G | G | A | G | U | A | U | U | G | U |
| AM920378 | U | U | C | G | ~ | G | A | A | C | U | U | G | G | G | U | G | G | A | G | U | A | U | U | G | U |
| X75763 | G | U | C | G | C | G | A | A | C | U | U | G | G | A | U | G | G | A | G | U | A | U | U | G | U |
| X74753 | U | U | C | G | C | G | A | A | C | U | U | G | G | G | U | G | A | A | G | U | A | U | U | G | U |
| X79495 | U | U | C | G | C | G | A | A | C | U | U | G | G | G | U | G | G | A | G | U | A | U | U | G | U |
| X79497 | U | U | C | G | G | G | A | A | C | U | U | G | G | G | C | G | G | A | G | U | A | U | U | G | U |
| AJ853450 | U | U | C | G | U | G | A | A | C | U | U | G | G | G | U | G | G | A | G | U | A | U | U | G | U |
| AJ853451 | U | U | C | G | U | G | A | A | C | U | U | G | G | G | U | G | G | A | G | U | A | U | U | G | U |
| AJ549234 | G | C | U | C | U | A | G | G | C | U | U | G | G | G | C | G | G | A | G | U | A | U | G | U | U |
| AJ549233 | G | U | A | U | C | A | G | A | C | U | U | G | G | G | C | A | G | A | G | U | A | U | G | U | U |
| AJ549232 | G | A | U | U | C | G | U | A | G | U | U | G | G | G | C | A | G | A | G | U | A | U | G | U | U |
| X70705 | G | U | C | G | C | G | ~ | A | C | U | U | G | G | G | U | G | G | A | G | U | A | U | G | G | U |
| KRA2_A | A | U | C | U | U | G | C | U | A | C | U | G | A | U | C | G | G | U | U | C | C | C | C | C | C |
| TIS1_A | A | U | C | U | U | G | C | U | A | C | U | G | A | U | C | G | G | U | U | C | C | C | C | C | C |
| WRH5_B | C | U | C | U | U | G | A | A | C | A | U | U | C | G | C | G | G | A | U | U | A | U | C | A | C |
| GTH1_B | C | U | C | U | C | G | A | A | C | A | U | U | C | G | C | G | G | A | U | U | A | U | C | A | C |
| MRT4A_C | C | U | C | U | U | G | A | A | C | A | U | U | U | G | C | G | G | A | U | A | A | U | C | G | C |
| TCA2_C | G | U | C | U | C | G | A | A | C | A | U | U | U | G | C | G | G | A | U | U | A | U | C | G | C |
| AJ428076 | U | U | C | U | U | G | A | A | C | A | U | U | U | G | C | G | G | A | U | U | A | U | C | G | C |
| WNII3A_D | C | U | C | U | U | G | A | A | C | A | U | A | C | G | U | G | G | A | U | U | A | U | C | C | C |
| SNG1_D | C | U | C | U | U | G | A | A | C | A | U | A | C | G | C | G | G | A | U | U | A | U | C | C | C |
| TRS7_E | G | U | C | C | A | G | G | C | G | A | U | U | C | G | C | G | G | C | U | G | A | U | C | U | C |
| ZIP2_F | G | C | C | C | C | G | A | A | U | A | A | U | C | G | C | U | G | C | U | ~ | A | U | C | U | C |
| AN3A_G | G | U | G | A | C | U | U | A | C | A | A | U | C | G | C | G | G | C | U | G | A | U | C | U | C |
| IGH2_H | G | G | U | G | C | C | G | A | C | G | C | U | C | C | G | G | C | C | U | ~ | A | U | C | U | C |

Table 5 List of NHS found for the genus *Spirogyra* and clades (continued)

| **Number** |  |  |  |  |  |  |  |  |  |  |  |  |  |  |  |  |  |  |  |  |  |  |  |  |  |  |  |  |  |  |  |  |  |  |  |
| --- | --- | --- | --- | --- | --- | --- | --- | --- | --- | --- | --- | --- | --- | --- | --- | --- | --- | --- | --- | --- | --- | --- | --- | --- | --- | --- | --- | --- | --- | --- | --- | --- | --- | --- | --- |
| *Spirogyra* |  | 58 | 59 | 60 |  | 62 | 63 | 64 | 65 |  |  |  | 69 | 70 | 71 | 72 | 73 | 74 | 75 |  |  | 78 | 79 | 80 | 81 |  |  |  | 85 | 86 |  | 88 |  |  |  |
| Clade A |  |  |  |  | 61 |  |  |  |  | 66 | 67 | 68 |  |  |  |  |  |  |  | 76 |  |  |  |  |  |  |  |  |  |  |  |  | 89 |  |  |
| Clade B |  |  |  |  |  |  |  |  |  |  |  |  |  |  |  |  |  |  |  |  |  |  |  |  |  |  | 83 | 84 |  |  |  |  |  | 90 |  |
| Clade C |  |  |  |  |  |  |  |  |  |  |  |  |  |  |  |  |  |  |  |  |  |  |  |  |  |  |  |  |  |  |  |  |  |  |  |
| Clade D | 57 |  |  |  |  |  |  |  |  |  |  |  |  |  |  |  |  |  |  |  |  |  |  |  |  |  |  |  |  |  | 87 |  |  |  |  |
| Clade E |  |  |  |  |  |  |  |  |  |  |  |  |  |  |  |  |  |  |  |  |  |  |  |  |  |  |  |  |  |  |  |  |  |  |  |
| Clade F |  |  |  |  |  |  |  |  |  |  |  |  |  |  |  |  |  |  |  |  |  |  |  |  |  | 82 |  |  |  |  |  |  |  |  | 91 |
| Clade G |  |  |  |  |  |  |  |  |  |  |  |  |  |  |  |  |  |  |  |  |  |  |  |  |  |  |  |  |  |  |  |  |  |  |  |
| Clade H |  |  |  |  |  |  |  |  |  |  |  |  |  |  |  |  |  |  |  |  | 77 |  |  |  |  |  |  |  |  |  |  |  |  |  |  |
| **Position in** | 0 | 1 | 1 | 1 | 1 | 1 | 1 | 1 | 1 | 1 | 1 | 1 | 1 | 1 | 1 | 1 | 1 | 1 | 1 | 1 | 1 | 1 | 1 | 1 | 1 | 1 | 1 | 1 | 1 | 1 | 1 | 1 | 1 | 1 | 1 |
| **Alignment** | 9 | 0 | 0 | 0 | 0 | 1 | 1 | 1 | 1 | 1 | 1 | 1 | 1 | 1 | 1 | 1 | 2 | 2 | 2 | 2 | 2 | 2 | 3 | 3 | 3 | 4 | 4 | 4 | 4 | 4 | 4 | 5 | 5 | 5 | 5 |
|  | 8 | 5 | 7 | 8 | 9 | 0 | 0 | 0 | 0 | 1 | 1 | 4 | 4 | 4 | 4 | 5 | 3 | 6 | 6 | 8 | 9 | 9 | 0 | 2 | 3 | 1 | 5 | 6 | 6 | 7 | 7 | 4 | 5 | 6 | 8 |
|  | 1 | 1 | 9 | 5 | 2 | 0 | 1 | 2 | 4 | 6 | 8 | 0 | 1 | 4 | 5 | 7 | 9 | 1 | 9 | 9 | 1 | 3 | 4 | 3 | 3 | 9 | 4 | 0 | 2 | 4 | 7 | 2 | 1 | 3 | 4 |
| **Helix number** | 25 | B27 | 27 | J27-28 | J27-28 | J28-29 | 29 | 29 | B29 | 29 | 29 | B29 | 29 | B29 | 29 | J30-28 | 34 | L35 | J35-36 | B37 | B37 | B37 | L37 | 37 | 38 | 43 | 43 | J43-44 | 44 | 44 | J44-38 | J34-45 | B45 | J45-46 | 46 |
| without : helix, J: link, L: endloop, B: buldge | | | | | |  |  |  |  |  |  |  |  |  |  |  |  |  |  |  |  |  |  |  |  |  |  |  |  |  |  |  |  |  |  |
| D13324 | A | U | U | A | A | U | A | G | G | G | U | C | A | U | U | A | C | A | A | G | G | G | U | U | G | C | G | G | U | A | U | C | U | U | G |
| X74000 | A | U | U | A | A | U | A | G | G | G | U | C | A | U | U | A | C | A | A | G | G | G | U | U | G | C | G | G | U | A | U | C | U | U | G |
| AF115438 | A | U | U | A | A | U | A | G | G | ~ | U | C | A | U | U | A | C | A | A | G | A | G | U | U | G | C | G | A | C | G | U | C | U | U | A |
| AF115437 | A | U | U | A | A | U | A | G | G | G | U | C | A | U | U | A | C | A | A | G | A | G | U | U | G | C | G | A | U | G | U | C | U | C | A |
| AF115442 | A | U | U | A | A | U | A | G | G | ~ | ~ | C | A | U | U | A | C | A | A | G | A | G | U | U | G | C | G | A | C | G | U | C | U | U | A |
| X91346 | A | U | U | A | A | U | A | G | G | G | U | C | A | U | U | A | C | A | A | G | A | G | U | U | G | C | G | A | U | A | U | C | U | C | A |
| AF115440 | A | U | U | A | A | U | A | G | G | G | U | C | A | U | U | A | C | A | A | G | A | G | U | U | G | C | G | A | C | G | U | C | U | U | A |
| X79496 | A | U | U | A | A | U | A | G | G | ~ | U | C | A | U | U | A | C | A | A | G | A | G | U | U | G | C | G | A | C | G | U | C | U | U | A |
| X77452 | A | U | U | A | A | U | A | G | G | ~ | ~ | C | A | U | U | A | C | A | A | G | A | G | U | U | G | C | G | A | C | G | U | C | U | U | A |
| X74752 | A | U | U | A | A | U | A | G | G | ~ | ~ | C | A | U | U | A | C | A | A | G | A | G | U | U | G | C | G | A | C | G | U | C | U | U | A |
| AF115439 | A | U | U | A | A | U | A | G | G | G | U | C | A | U | U | A | C | A | A | G | A | G | U | U | G | C | G | A | U | A | U | C | U | U | G |
| AM920378 | A | U | U | A | A | U | A | G | G | ~ | U | C | A | U | U | A | C | A | A | G | A | G | U | U | G | C | G | A | C | G | U | C | U | U | A |
| X75763 | A | U | U | A | A | U | A | G | G | G | U | C | A | U | U | A | C | A | A | G | A | G | U | U | G | C | G | A | U | A | U | C | U | U | G |
| X74753 | A | U | U | A | A | U | A | G | G | G | U | C | A | U | U | A | C | A | A | G | A | G | U | U | G | C | G | A | U | A | U | C | U | ~ | A |
| X79495 | A | U | U | A | A | U | A | G | G | G | U | C | A | U | U | A | C | A | A | G | A | G | U | U | G | C | G | A | U | A | U | C | U | U | G |
| X79497 | A | U | U | A | A | U | A | G | G | ~ | U | C | A | U | U | A | C | A | A | G | A | G | U | U | G | C | G | A | C | G | U | C | U | U | A |
| AJ853450 | A | U | U | A | A | U | A | G | G | G | U | C | A | U | U | A | C | A | A | G | A | G | U | U | G | C | G | A | U | A | U | C | U | U | G |
| AJ853451 | A | U | U | A | A | U | A | G | G | G | U | C | A | U | C | A | C | A | A | G | A | G | U | U | G | C | G | A | U | A | U | C | U | U | G |
| AJ549234 | G | U | U | A | A | U | A | G | G | G | U | C | A | U | U | A | C | A | A | G | A | G | U | U | G | C | G | A | U | G | U | C | U | U | G |
| AJ549233 | G | U | U | A | A | U | A | G | G | G | G | C | A | U | U | A | C | A | A | G | A | G | U | U | G | C | G | A | U | A | U | C | U | U | G |
| AJ549232 | G | U | U | A | A | U | A | G | G | G | U | C | A | U | U | A | C | A | A | G | A | G | U | U | G | C | G | A | C | G | U | C | U | U | G |
| X70705 | A | U | U | A | A | U | A | G | G | G | U | C | A | U | C | A | C | A | A | G | A | G | U | U | G | C | G | A | U | A | U | C | U | U | G |
| KRA2_A | A | G | C | G | U | C | C | C | A | A | C | A | G | C | G | G | G | G | U | U | A | C | C | C | A | C | G | A | G | C | U | A | C | U | G |
| TIS1_A | A | G | C | G | U | C | C | C | A | A | C | A | G | C | G | G | G | G | U | U | A | C | C | C | A | C | G | A | G | C | U | A | C | U | G |
| WRH5_B | A | G | C | G | A | C | C | C | A | G | A | C | G | C | G | G | G | G | U | G | G | C | C | C | A | C | A | U | G | C | U | A | U | A | G |
| GTH1_B | A | G | C | G | A | C | C | C | A | G | A | C | G | C | G | G | G | G | U | G | G | C | C | C | A | C | A | U | G | C | U | A | U | A | G |
| MRT4A_C | A | G | C | G | A | C | C | C | A | G | A | C | G | C | G | G | G | G | U | G | G | C | C | C | A | G | G | G | G | C | U | A | U | C | G |
| TCA2_C | C | G | C | G | A | C | C | C | A | G | A | C | G | C | G | G | G | G | U | G | G | C | C | C | A | G | G | G | G | C | U | A | U | C | G |
| AJ428076 | A | G | C | G | A | C | C | C | A | G | A | C | G | C | G | G | G | G | U | G | G | C | C | C | A | G | G | G | G | C | U | A | U | C | G |
| WNII3A_D | U | G | C | G | A | C | C | C | A | G | A | C | G | C | G | G | G | G | U | G | G | C | C | C | A | C | G | G | G | C | C | A | U | U | G |
| SNG1_D | U | G | C | G | A | C | C | C | A | G | A | C | G | C | G | G | G | G | U | G | G | C | C | C | A | C | G | G | G | C | C | A | U | U | G |
| TRS7_E | U | G | C | G | A | C | C | C | A | G | A | C | G | C | G | G | G | G | U | G | G | C | C | C | A | C | G | G | G | C | U | A | U | C | G |
| ZIP2_F | C | G | C | G | A | C | C | C | A | G | A | C | G | C | G | G | G | G | U | G | G | C | C | C | A | U | G | G | G | C | U | A | G | C | U |
| AN3A_G | C | G | C | G | A | C | C | C | A | G | A | C | G | C | G | G | G | G | U | G | G | C | C | C | A | C | G | G | G | C | U | A | G | C | G |
| IGH2_H | C | G | C | G | A | C | C | C | A | G | A | C | G | C | G | G | G | G | U | G | C | C | C | C | A | C | G | G | G | C | U | A | G | C | G |

Table 5 List of NHS found for the genus *Spirogyra* and clades (continued)

| **Number** |  |  |  |  |  |  |  |  |  |  |  |  |  |  |  |  |  |  |
| --- | --- | --- | --- | --- | --- | --- | --- | --- | --- | --- | --- | --- | --- | --- | --- | --- | --- | --- |
| *Spirogyra* |  | 93 | 94 |  | 96 | 97 | 98 | 99 | 100 | 101 |  |  | 104 |  |  | 107 |  | 109 |
| Clade A | 92 |  |  |  |  |  |  |  |  |  |  | 103 |  |  | 106 |  |  |  |
| Clade B |  |  |  |  |  |  |  |  |  |  | 102 |  |  |  |  |  |  |  |
| Clade C |  |  |  |  |  |  |  |  |  |  |  |  |  | 105 |  |  | 108 |  |
| Clade D |  |  |  |  |  |  |  |  |  |  |  |  |  |  |  |  |  |  |
| Clade E |  |  |  |  |  |  |  |  |  |  |  |  |  |  |  |  |  |  |
| Clade F |  |  |  |  |  |  |  |  |  |  |  |  |  |  |  |  |  |  |
| Clade G |  |  |  | 95 |  |  |  |  |  |  |  |  |  |  |  |  |  |  |
| Clade H |  |  |  |  |  |  |  |  |  |  |  |  |  |  |  |  |  |  |
| **Position** | 1 | 1 | 1 | 1 | 1 | 1 | 1 | 1 | 1 | 1 | 1 | 1 | 1 | 1 | 1 | 1 | 1 | 1 |
| **in Alignment** | 6 | 6 | 6 | 6 | 6 | 6 | 6 | 6 | 6 | 7 | 7 | 7 | 7 | 7 | 7 | 7 | 7 | 8 |
|  | 1 | 2 | 2 | 3 | 3 | 6 | 7 | 8 | 8 | 2 | 3 | 5 | 6 | 6 | 7 | 7 | 8 | 0 |
|  | 9 | 0 | 2 | 3 | 5 | 5 | 5 | 0 | 4 | 9 | 8 | 9 | 0 | 6 | 2 | 8 | 9 | 6 |
| **Helix number** | 47 | 47 | L47 | L47 | 47 | 48 | 48 | 48 | J48-32 | B49 | B49 | B49 | 49 | B49 | B49 | L49 | B49 | 49 |
| without : helix, J: link, L: endloop, B: buldge | | | | | |  |  |  |  |  |  |  |  |  |  |  |  |  |
| D13324 | A | U | A | U | A | A | U | U | U | U | C | ~ | G | G | C | G | C | C |
| X74000 | A | U | A | U | A | A | U | U | U | U | C | ~ | G | G | C | ~ | C | C |
| AF115438 | A | U | A | U | A | A | U | U | U | U | C | C | G | G | C | G | U | C |
| AF115437 | A | U | A | U | A | A | U | U | U | U | C | C | G | G | C | C | C | C |
| AF115442 | A | U | A | U | A | A | U | U | U | U | C | C | G | G | C | G | U | C |
| X91346 | A | U | A | U | A | A | U | U | U | U | C | U | G | G | C | G | C | A |
| AF115440 | A | U | A | U | A | A | U | U | U | U | C | C | G | G | C | G | C | C |
| X79496 | A | U | A | U | A | A | U | U | U | U | C | C | U | G | C | G | U | C |
| X77452 | A | U | A | U | A | A | U | U | U | U | C | C | G | ~ | C | G | U | C |
| X74752 | A | U | A | U | A | A | U | U | U | U | C | C | G | G | C | G | U | C |
| AF115439 | A | U | A | U | A | A | U | U | U | U | C | U | G | G | C | G | U | C |
| AM920378 | A | U | A | U | A | A | U | U | U | U | C | C | G | G | C | G | U | C |
| X75763 | A | U | A | U | A | A | U | U | U | U | C | U | G | G | C | G | U | C |
| X74753 | A | U | A | U | A | A | U | U | U | U | C | C | G | G | C | G | C | A |
| X79495 | A | U | A | U | A | A | U | U | U | U | C | U | G | G | C | G | U | C |
| X79497 | A | U | A | U | A | A | U | U | U | U | C | C | G | G | C | G | U | C |
| AJ853450 | A | U | A | U | A | A | U | U | U | U | C | U | G | G | C | G | U | C |
| AJ853451 | A | U | A | U | A | A | U | U | U | U | C | U | G | G | C | G | U | C |
| AJ549234 | A | U | A | U | A | A | U | U | U | U | C | C | G | G | C | G | U | C |
| AJ549233 | A | U | A | U | A | A | U | U | U | U | C | C | A | G | C | G | U | C |
| AJ549232 | A | U | A | U | A | A | U | U | U | U | C | C | A | G | C | G | U | C |
| X70705 | A | U | A | U | A | A | U | U | U | U | C | U | G | G | C | G | C | C |
| KRA2_A | C | C | C | U | G | C | G | C | C | A | C | C | C | G | A | A | G | U |
| TIS1_A | C | C | C | U | G | C | G | C | C | A | C | C | C | G | A | A | G | U |
| WRH5_B | A | C | C | U | G | C | G | C | C | A | U | C | C | G | C | A | G | U |
| GTH1_B | A | C | C | U | G | C | G | C | C | A | U | C | C | G | C | A | G | U |
| MRT4A_C | A | C | C | U | G | C | G | C | C | A | C | C | C | A | C | A | A | U |
| TCA2_C | A | C | C | U | G | C | G | C | C | A | C | C | C | A | C | A | A | U |
| AJ428076 | A | C | C | U | G | C | G | ~ | C | A | C | C | C | G | C | A | A | U |
| WNII3A_D | A | C | C | U | G | C | G | C | C | A | C | C | C | G | C | A | G | U |
| SNG1_D | A | C | C | U | G | C | G | C | C | A | C | C | C | G | C | A | G | U |
| TRS7_E | A | C | C | U | G | C | G | C | C | A | C | C | C | G | C | A | G | U |
| ZIP2_F | A | C | C | C | G | C | G | C | C | A | C | C | C | G | C | A | G | U |
| AN3A_G | A | C | C | G | G | C | G | C | C | A | C | C | C | G | C | A | G | U |
| IGH2_H | A | C | C | C | G | C | G | C | C | A | C | C | C | G | C | A | C | U |
