# Supplementary material for: Hidden genetic diversity in the green alga Spirogyra (Zygnematophyceae, Streptophyta)
Source: BMC Evol Biol. 2012 Jun 1;12:77. doi: 10.1186/1471-2148-12-77 (PMC3527229; doi:10.1186/1471-2148-12-77)
Supplement: Additional file 2 — Table S2. Comparison of the maximum likelihood tree (Spirogyra alignment) with user defined trees by AU, KH, SH and weighted SH. Trees significantly worse than the best trees at p≤ 0.05 are indicated by grey highlighting. [file 1471-2148-12-77-S2.doc]

Table 6: Origin of isolates

| **Abbreviation** | **Origin** | **Source** | **Accession No.** | **Clade** | **Intron** | **represented by** | **GPS Coordinates** | |
| --- | --- | --- | --- | --- | --- | --- | --- | --- |
| 7074 |  | ASW | JQ290232 | E | - | 7074 | N.A. | N.A. |
| 7075 |  | ASW | JQ290233 | D-E2 | - | 7075 | N.A. | N.A. |
| 7076 |  | ASW | JQ239109 | D | + | 7076 | N.A. | N.A. |
| 7208 |  | ASW | JQ239078 | C | + | 7208 | N.A. | N.A. |
| 071015-1e | Fish pond Franzen | AUT | JQ290230 | F | - | BGHIII1 | 15° 23' 49" | 48° 37' 07" |
| 1020A1 | Pond 1020, Vienna | AUT | JQ290231 | D | + | TIS3 | 16° 21' 09" | 48° 09' 21" |
| AB1 | between Ahrensdorf & Behrensdorf | GER | JQ239111 | D | + | AB4 | 14° 03' 32" | 52° 10' 52" |
| AB4 | between Ahrensdorf & Behrensdorf | GER | JQ239110 | D | + | AB4 | 14° 03' 32" | 52° 10' 52" |
| ADA1 | Alte Donau | AUT | JQ290234 | D-E2 | - | 7075 | 16° 24' 29" | 48° 14' 58" |
| ADA2 | Alte Donau | AUT | JQ290235 | D-E2 | - | 7075 | 16° 24' 29" | 48° 14' 58" |
| ADA6 | Alte Donau | AUT | JQ290236 | D-E2 | - | 7075 | 16° 24' 29" | 48° 14' 58" |
| AN3A | Alte Naufahrt | AUT | JQ239122 | G | - | AN3A | 16° 28' 15" | 48° 11' 46" |
| AN4B | Alte Naufahrt | AUT | JQ290237 | H | - | IGH2 | 16° 28' 15" | 48° 11' 46" |
| BEA1 | Benda pond | AUT | JQ239098 | D | + | KVW2 | 16° 20' 58" | 48° 09' 11" |
| BGHC1 | botanical garden Hamburg chinese pond | GER | JQ290238 | H | - | GRS1 | 09° 51' 40" | 53° 33' 52" |
| BGHII4 | Botanical garden Hamburg near entrance | GER | JQ239049 | C | + | REC2 | 09° 51' 37" | 53° 33' 43" |
| BGHIII1 | botanical garden Hamburg pond for halophilic plants | GER | JQ290239 | F | - | BGHIII1 | 09° 51' 36" | 53° 33' 47" |
| BGHIII2 | botanical garden Hamburg pond for halophilic plants | GER | JQ290240 | F | - | BGHIII1 | 09° 51' 36" | 53° 33' 47" |
| BGHK1 | Botanical garden Hamburg limestone alpine area | GER | JQ239112 | D | + | BGHK1 | 09° 51' 36" | 53° 33' 50" |
| BGHK2 | Botanical garden Hamburg limestone alpine area | GER | JQ239113 | D | + | NES3 | 09° 51' 36" | 53° 33' 50" |
| BTSA1 | Pond Suessenbrunn | AUT | JQ239106 | D | + | VNE2 | 16° 29' 09" | 48° 16' 36" |
| BTSA3 | Pond Suessenbrunn | AUT | JQ290241 | H | - | SOT1A | 16° 29' 09" | 48° 16' 36" |
| DECA3 | Dechantlacke | AUT | JQ290242 | D-E2 | - | 7075 | 16° 28' 34" | 48° 11' 33" |
| DECA4 | Dechantlacke | AUT | JQ290243 | E | - | DECA4 | 16° 28' 34" | 48° 11' 33" |
| DRS1 | Dranser lake | GER | JQ290244 | F | - | BGHIII1 | 12° 37' 53" | 53° 10' 48" |
| DRS2 | Dranser lake | GER | JQ290245 | F | - | BGHIII1 | 12° 37' 53" | 53° 10' 48" |
| DRS3 | Dranser lake | GER | JQ239059 | C | + | MRT4A | 12° 37' 53" | 53° 10' 48" |
| EGR4 | drainage trench Reddelich | GER | JQ239065 | B | + | EGR4 | 11° 49' 52" | 54° 04' 38" |
| EIT4A | Eitzenberger pond | GER | JQ290246 | F | - | EIT4A | 11° 21' 50" | 47° 47' 11" |
| FBT1A | Fuchsbodenteich | AUT | JQ239114 | D | + | TIS3 | 15° 51' 45" | 48° 21' 54" |
| FBT3A | Fuchsbodenteich | AUT | JQ290247 | G | - | FBT3A | 15° 51' 45" | 48° 21' 54" |
| FIT3A | Filmteich | AUT | JQ239079 | B | + | FIT3A | 16° 24' 17" | 48° 09' 01" |
| GBT1 | gr Brunnerteich | AUT | JQ239064 | B | + | GBT1 | 16° 18' 30" | 48° 07' 00" |
| GBT3 | gr Brunnerteich | AUT | JQ239092 | B | + | GBT3 | 16° 18' 30" | 48° 07' 00" |
| GRS1 | Hamburg Großensee (pier) | GER | JQ239080 | H | - | GRS1 | 10° 20' 51" | 53° 36' 46" |
| GRS2 | Hamburg Großensee (pier) | GER | JQ290248 | H | - | GRS1 | 10° 20' 51" | 53° 36' 46" |
| GSB2 | gr. Segeberger Lake | AUT | JQ239118 | D | + | GSB2 | 10° 20' 23" | 53° 57' 27" |
| GTH1 | garden pond Hamburg | GER | JQ239067 | B | + | GTH1 | 10° 08' 44" | 53° 37' 21" |
| GTH7 | garden pond Hamburg | GER | JQ239068 | B | + | GTH1 | 10° 08' 44" | 53° 37' 21" |
| GUS1 | Grubensee | AUT | JQ239096 | D | + | KVW2 | 13° 59' 40" | 52° 09' 30" |
| HRM4A | Rotmoos Lunz | AUT | JQ290249 | E | - | HRM4A | N.A. | N.A. |
| IGH2 | Industrial area Höltingbaum | GER | JQ239123 | H | - | IGH2 | 10° 11' 01" | 53° 36' 44" |
| IGH3 | Industrial area Höltingbaum | GER | JQ290250 | H | - | IGH2 | 10° 11' 01" | 53° 36' 44" |
| IGH4 | Industrial area Höltingbaum | GER | JQ290251 | H | - | IGH2 | 10° 11' 01" | 53° 36' 44" |
| KAT1A | Kastanienalleeteich | AUT | JQ290252 | H | - | SOT1A | 16° 20' 49" | 48° 09' 26" |
| KRA2 | watercourse between Krauswitz & Schlagnitz | GER | JQ239054 | A | + | KRA2 | 13° 52' 50" | 52° 01' 48" |
| KVW2 | Pond near traffic circle A19 Wittstock | GER | JQ239094 | D | + | KVW2 | 12° 27' 20" | 53° 09' 26" |
| KVW4 | Pond near traffic circle A19 Wittstock | GER | JQ239097 | D | + | KVW2 | 12° 27' 20" | 53° 09' 26" |
| KW1A | Kaiserwasser | AUT | JQ239089 | B | + | KWA3 | 16° 25' 29" | 48° 13' 54" |
| KW4A | Kaiserwasser | AUT | JQ239090 | B | + | KWA3 | 16° 25' 29" | 48° 13' 54" |
| KWA3 | Kaiserwasser | AUT | JQ239087 | B | + | KWA3 | 16° 25' 29" | 48° 13' 54" |
| LHS4 | Langhagensee | GER | JQ290253 | F | - | WIND3A | 12° 40' 31" | 53° 14' 53" |
| LHS5 | Langhagensee | GER | JQ290254 | F | - | WIND3A | 12° 40' 31" | 53° 14' 53" |

Table 6: Origin of isolates (continued)

| **Abbreviation** | **Origin** | **Source** | **Accession No.** | **Clade** | **Intron** | **represented by** | **GPS Coordinates** | |
| --- | --- | --- | --- | --- | --- | --- | --- | --- |
| LHS6 | Langhagensee | GER | JQ290255 | F | - | WIND3A | 12° 40' 31" | 53° 14' 53" |
| LHS8 | Langhagensee | GER | JQ290256 | B | + | LHS8 | 12° 40' 31" | 53° 14' 53" |
| LHS9 | Langhagensee | GER | JQ290257 | F | - | WIND3A | 12° 40' 31" | 53° 14' 53" |
| LL1 | local fire service pond Limsdorf | GER | JQ239056 | C | + | MRT4A | 14° 00' 51" | 52° 09' 34" |
| LL2 | local fire service pond Limsdorf | GER | JQ239057 | C | + | MRT4A | 14° 00' 51" | 52° 09' 34" |
| LSB4A | Langenschoenbichl | AUT | JQ239091 | B | + | KWA3 | 15° 59' 45" | 48° 19' 52" |
| LUA1 | Lunzer Untersee outflow | AUT | JQ290258 | F | - | WIND3A | 15° 02' 25" | 47° 51' 12" |
| LUBS1 | Lunzer lake, stone near boathouse | AUT | JQ290259 | E | - | DECA4 | 15° 03' 41" | 47° 51' 16" |
| M3A | St. Pölten | AUT | JQ239048 | C | + | M3A | N.A. | N.A. |
| M5A | St. Pölten | AUT | JQ239075 | B | + | M6A | N.A. | N.A. |
| M6A | St. Pölten | AUT | JQ239076 | B | + | M6A | N.A. | N.A. |
| M9A | St. Pölten | AUT | JQ239062 | C | + | MRT4A | N.A. | N.A. |
| M9B | St. Pölten | AUT | JQ239074 | B | + | M6A | N.A. | N.A. |
| MD2-1 | St. Pölten | AUT | JQ239060 | C | + | MRT4A | N.A. | N.A. |
| MDA1 | St. Pölten | AUT | JQ239061 | C | + | MRT4A | N.A. | N.A. |
| MIL10A | Millstaetter Lake | AUT | JQ290260 | H | - | GRS1 | 13° 31' 10" | 46° 48' 51" |
| MP9-1 | St. Pölten | AUT | JQ239069 | B | + | M6A | N.A. | N.A. |
| MP92-3 | St. Pölten | AUT | JQ239073 | B | + | M6A | N.A. | N.A. |
| MP92-6 | St. Pölten | AUT | JQ239072 | B | + | M6A | N.A. | N.A. |
| MRT4A | Mausrodlteich | AUT | JQ239058 | C | + | MRT4A | 15° 02' 52" | 47° 52' 25" |
| NES3 | Nebelsee | GER | JQ239119 | D | + | NES3 | 12° 39' 58" | 53° 14' 54" |
| NES4 | Nebelsee | GER | JQ239120 | D | + | NES3 | 12° 39' 58" | 53° 14' 54" |
| NLH5 | watercourse between Neu-Lübbenau & Hohenbrück | GER | JQ239084 | B | + | NLH5 | 13° 53' 38" | 52° 04' 57" |
| NLH6 | watercourse between Neu-Lübbenau & Hohenbrück | GER | JQ239063 | B | + | NLH6 | 13° 53' 38" | 52° 04' 57" |
| OROSIVA1 | Osor | HR | JQ239104 | D | + | SNG1 | 14° 23' 87" | 44° 41,732' |
| OSS5A | Ossiacher lake | AUT | JQ290283 | H | - | OSS5A | 13° 58' 51" | 46° 40' 37" |
| PANA1 | Panozzalacke | AUT | JQ239085 | B | + | PANA1 | 16° 29' 16" | 48° 10' 51" |
| PLO1 | Plötinsee, Rostock | GER | JQ239115 | D | + | TIS3 | 12° 58' 22" | 53° 13' 14" |
| REC2 | Meadow pond Rechlin | GER | JQ239051 | C | + | REC2 | 12° 45' 45" | 53° 18' 43" |
| REC3 | Meadow pond Rechlin | GER | JQ239105 | D | + | VNE2 | 12° 45' 45" | 53° 18' 43" |
| ROE2 | Pond near Rödel | GER | JQ290261 | F | - | EIT4A | 12° 35' 51" | 53° 23' 06" |
| ROE6 | Pond near Rödel | GER | JQ239093 | B | + | ROE6 | 12° 35' 51" | 53° 23' 06" |
| S1DD3A | St. Pölten | AUT | JQ239070 | B | + | M6A | N.A. | N.A. |
| S1Due2-2A | St. Pölten | AUT | JQ239071 | B | + | M6A | N.A. | N.A. |
| S1Due2A | St. Pölten | AUT | JQ239077 | B | + | M6A | N.A. | N.A. |
| SENA4 | Sengsee | GER | JQ239082 | B | + | SENA7 | 11° 18' 58" | 47° 46' 37" |
| SENA7 | Sengsee | GER | JQ239081 | B | + | SENA7 | 11° 18' 58" | 47° 46' 37" |
| SHS6A | Schilfhuettensee | GER | JQ290262 | F | - | WIND3A | 11° 19' 02" | 47° 46' 26" |
| SKA1 | Kritzendorf | AUT | JQ290263 | F | - | WIND3A | 16° 18' 48" | 48° 20' 06" |
| SKA3 | Kritzendorf | AUT | JQ290264 | F | - | WIND3A | 16° 18' 48" | 48° 20' 06" |
| SKR1 | pond near Kröpelin, Rostock | GER | JQ290265 | F | - | WIND3A | 11° 48' 45" | 54° 04' 25" |
| SKR2 | pond near Kröpelin, Rostock | GER | JQ290266 | F | - | WIND3A | 11° 48' 45" | 54° 04' 25" |
| SMS2 | Scharmützelsee | GER | JQ239066 | B | + | SMS2 | 14° 00' 50" | 52° 12' 49" |
| SNG1 | Marsh north Großensee | GER | JQ239101 | D | + | SNG1 | 10° 21' 27" | 53° 37' 51" |
| SNG2 | Marsh north Großensee | GER | JQ239100 | D | + | SNG1 | 10° 21' 27" | 53° 37' 51" |
| SNG3 | Marsh north Großensee | GER | JQ239103 | D | + | SNG1 | 10° 21' 27" | 53° 37' 51" |
| SNG5 | Marsh north Großensee | GER | JQ239102 | D | + | SNG1 | 10° 21' 27" | 53° 37' 51" |
| SOT1A | Stierofenteich | AUT | JQ290267 | H | - | SOT1A | 16° 20' 57" | 48° 09' 23" |
| SOTA1 | Stierofenteich | AUT | JQ290268 | H | - | SOT1A | 16° 20' 57" | 48° 09' 23" |
| SPS3 | Springsee | GER | JQ290269 | D-E2 | - | 7075 | 13° 59' 19" | 52° 10' 20" |
| TCA2 | Canow fish farming | GER | JQ239050 | C | + | REC2 | 12° 53' 23" | 53° 11' 32" |
| TIS1 | Tiefensee | GER | JQ239052 | A | + | TIS1 | 13° 59' 22" | 52° 09' 23" |
| TIS3 | Tiefensee | GER | JQ239117 | D | + | TIS3 | 13° 59' 22" | 52° 09' 23" |

Table 6: Origin of isolates (continued)

| **Abbreviation** | **Origin** | **Source** | **Accession No.** | **Clade** | **Intron** | **represented by** | **GPS Coordinates** | |
| --- | --- | --- | --- | --- | --- | --- | --- | --- |
| TIS4 | Tiefensee | GER | JQ239116 | D | + | TIS3 | 13° 59' 22" | 52° 09' 23" |
| TOR4 | Torsee | GER | JQ239083 | B | + | TOR4 | 13° 39' 56" | 52° 14' 24" |
| TRS7 | Tristacher Lake | AUT | JQ239121 | E | - | TRS7 | 12° 47' 40" | 46° 48' 29" |
| UMW4A | Lower Muehlwasser | AUT | JQ239055 | A | + | UMW4A | 16° 27' 20" | 48° 12' 48" |
| UTEX LB 1984 |  | UTEX | JQ239053 | C | + | UTEX1984 | N.A. | N.A. |
| UTEX1273 Sp. crassispina |  | UTEX | JQ290270 | H | - | UTEX1273 | N.A. | N.A. |
| UTEX1683 Sp. occidentalis |  | UTEX | JQ290271 | H | - | UTEX1683 | N.A. | N.A. |
| UTEX1742 Sp. juergensis |  | UTEX | JQ290272 | D-E1 | - | UTEX1742 | N.A. | N.A. |
| UTEX1745 Sp. liana |  | UTEX | JQ290273 | E-F | - | UTEX1745 | N.A. | N.A. |
| UTEX1746 Sp. pratensis |  | UTEX | JQ290275 | G | - | UTEX1746 | N.A. | N.A. |
| UTEX2495 Sp. maxima |  | UTEX | JQ290274 | C | + | UTEX2495 | N.A. | N.A. |
| VNE2 | accretion zone Neversdorfer lake | GER | JQ239107 | D | + | VNE2 | 10° 15' 31" | 53° 51' 56" |
| W4A | bog-drain, Windtal | I | JQ290276 | F | - | WIND3A | N.A. | N.A. |
| WB5A | Wienerberg pond2 | AUT | JQ239099 | D | + | KVW2 | 16° 20' 49" | 48° 09' 43" |
| WBA2 | rivulet, Wienerberg | AUT | JQ290277 | H | - | WBA2 | 16° 21' 06" | 48° 09' 29" |
| WBA3 | rivulet, Wienerberg | AUT | JQ290278 | H | - | IGH2 | 16° 21' 06" | 48° 09' 29" |
| WBT2A | Wienerberg pond2 | AUT | JQ290279 | H | - | GRS1 | 16° 20' 49" | 48° 09' 43" |
| WIND3A | bog-drain, Windtal | I | JQ290280 | F | - | WIND3A | N.A. | N.A. |
| WNII3A | local fire service pond Gerasdorf | AUT | JQ239108 | D | + | WNII3A | 16° 27' 58" | 48° 18' 00" |
| WNII4A | local fire service pond Gerasdorf | AUT | JQ239086 | B | + | PANA1 | 16° 27' 58" | 48° 18' 00" |
| WNIII2A | Pond recreation area Seeschlacht | AUT | JQ239095 | D | + | KVW2 | 16° 21' 49" | 48° 17' 56" |
| WOE4A | western pond near oilharbor | AUT | JQ290281 | D-E2 | - | 7075 | 16° 31' 07" | 48° 10' 00" |
| WRH5 | Wandse detention reservoir | GER | JQ239088 | B | + | KWA3 | 10° 11' 07" | 53° 37' 17" |
| ZIP1 | Zipke | GER | JQ290282 | F | - | ZIP2 | 12° 47'02" | 54° 20' 30" |
| ZIP2 | Zipke | GER | JQ239124 | F | - | ZIP2 | 12° 47'02" | 54° 20' 30" |
