# Supplementary material for: Hidden genetic diversity in the green alga Spirogyra (Zygnematophyceae, Streptophyta)
Source: BMC Evol Biol. 2012 Jun 1;12:77. doi: 10.1186/1471-2148-12-77 (PMC3527229; doi:10.1186/1471-2148-12-77)
Supplement: Additional file 3 — Table S3. Results of the Relative Rate Test carried out in GRate [56]; using only unambiguously aligned positions of all sequences used in this study; not significant: N.S. (p > 0.05; relative rates not significantly different at 5% level). Asterisks: p = 0.05 > * > 0.01 > ** > 0.005 > *** (relative rates significantly different). [file 1471-2148-12-77-S3.doc]

Additional file 3: Table 6: Results of the Relative Rate Test carried out in GRate [56]; using only unambiguously aligned positions of all sequences used in this study; not significant: N.S. (p > 0.05; relative rates not significantly different at 5% level). Asterisks: p = 0.05 > * > 0.01 > ** > 0.005 > *** (relative rates significantly different).
